# Single‐Molecule Forster Resonance Energy Transfer With a Minimalistic 3D‐Printed Setup and Dyes in the Blue‐Green Spectral Region

**DOI:** 10.1002/cphc.202500909

**Published:** 2026-04-30

**Authors:** Gabriel G. Moya Muñoz, Jorge R. Luna Piedra, Pazit Con, Mostofa Ataur Rohman, Siyu Lu, Thomas‐Otavio Peulen, Thorben Cordes

**Affiliations:** ^1^ Biophysical Chemistry, Department of Chemistry and Chemical Biology Technische Universität Dortmund Dortmund Germany; ^2^ Physical and Synthetic Biology, Faculty of Biology Ludwig‐Maximilians‐Universität München Planegg‐Martinsried Germany; ^3^ FluoBrick Solutions GmbH Berlin Germany; ^4^ Department of Poultry and Aquaculture Institute of Animal Sciences, Agricultural Research Organization Volcani Center Rishon LeZion Israel

## Abstract

Förster Resonance Energy Transfer (FRET) is a powerful technique for the detection and characterization of biomolecular interactions and conformational changes with subnanometer spatial resolution and a temporal resolution down to the timescale of fluorescence. While the technique is widely adopted in structural biology and biophysics, the evolution of single‐molecule FRET has led to experimental setups with sophisticated optical layouts, multilaser excitation schemes, and time‐resolved detection electronics. We here present an accessible alternative toward single‐molecule FRET based on Brick‐MIC, a recently introduced three‐dimensional (3D)‐printed microspectroscopy platform. The FRET‐Brick uses continuous‐wave excitation at 488 nm with a minimal set of optomechanical components and photomultiplier detectors (PMTs). With this, we were able to significantly reduce the setup complexity retaining single‐molecule sensitivity with dyes matching the sensitivity of PMTs. To maximize the photon output of Alexa488, ATTO488 (donors), Alexa555, ATTO542, and Cy3B (acceptors), we introduce ferrocene derivatives as photostabilizers that increase both dye brightness and remove dark‐states. We benchmark the performance of the FRET‐Brick with fluorophore‐labeled oligonucleotide reference structures also in comparison to accessible volume simulations, and by detecting conformational changes in bacterial substrate‐binding proteins. Our work demonstrates that qualitative and quantitative single‐molecule FRTE (smFRET) measurements are possible with the minimalistic and cost‐effective FRET‐Brick.

## Graphical abstract

Single‐molecule FRET enables nanometer‐scale insights into biomolecular structure and dynamics but often requires complex instrumentation. We present the FRET‐Brick, a minimalistic, 3D‐printed microspectroscopy platform using 488 nm continuous‐wave excitation and PMT detection. Enhanced by ferrocene photostabilizers, the system achieves high sensitivity, validated with nucleic acid and protein standards. The study demonstrates accessible, cost‐effective and quantitative smFRET measurements for biological applications.

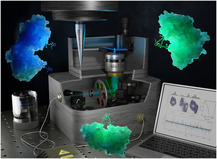

## 1 Introduction

Single‐molecule Förster Resonance Energy Transfer (smFRET) has become an established technique for studying biomolecular interactions and conformational dynamics of biomolecules in vitro and in vivo [1, 2, 3, 4, 5, 6, 7, 8, 9, 10, 11]. Over time, smFRET instrumentation has evolved to include multilaser excitation schemes, complex timing electronics, and sophisticated optical arrangements to improve its sensitivity, but also the temporal and spatial resolution [12, 13, 14, 15]. Multiparameter fluorescence detection (MFD) [16, 17], alternating‐laser excitation (ALEX [12, 18, 19]), and pulsed interleaved excitation (PIE [13]) have significantly advanced smFRET by enabling precise characterization of biomolecular labeling with donor and acceptor dyes and the availability of multidimensional data sets and in‐depth information on the biomolecular system [13, 18, 19]. These allow for the correction of setup‐dependent artifacts such as spectral cross talk, differences in quantum yields between donor and acceptor dyes, and the wavelength‐dependent detection efficiencies of the optical system [6]. Correcting for these is a requirement to convert FRET efficiency into accurate interdye distance (e.g., for integrative structural modeling) [12], but also to identify conformational dynamics and structural ensembles. In essence, many technical developments focused on improving the sensitivity, precision and accuracy, and the information content of smFRET as a quantitative nanoscale ruler [1, 2].

Together with advancements in the standardization of measurement and analysis routines, smFRET is by now an established tool for mechanistic biophysical studies but also a quantitative method in structural biology [1, 2, 3, 4, 5, 6, 7, 8, 9, 10, 11, 20, 21, 22, 23, 24, 25, 26, 27, 28]. The required complexity, but also the costs related to the purchase of an smFRET instrument, raised the bar for nonspecialized laboratories to enter the field and independently conduct smFRET experiments. Indeed, many applied users of smFRET have the goal to only qualitatively visualize ligand binding, domain reorientation, or conformational switching. These studies often follow an iterative process: comparing apparent FRET efficiencies under different biochemical conditions, identifying contrasts, and inferring mechanistic insights. For that purpose, measurement of relative FRET efficiency changes is sufficient and extracting absolute distances (or other spectroscopic parameters such as lifetime or anisotropy) is not strictly required [20, 21, 22, 23, 24, 25, 26, 27, 28]. Thus, setups with reduced complexity would suffice for such questions since they allow to address (most) relevant aspects of the research question.

Recent efforts from our lab and others [29, 30, 31, 32, 33, 34, 35, 36] demonstrated the power of compact and affordable microscopy designs suitable for advanced optical imaging [29, 31, 33, 34, 35]. This open‐source microscopy direction included applications such as smFRET [29] and superresolution microscopy [29, 34, 35] with three‐dimensional (3D)‐printed scaffolds [29, 30, 33] and simplified optic layouts. Building on this foundation, we here present a minimalistic smFRET extension of our Brick‐MIC platform [29] that greatly reduces instrumental complexity and costs. The confocal FRET‐Brick is capable to detect individual diffusing molecules and uses a simple 488 nm continuous‐wave (cw) excitation laser, detection via photomultiplier tubes (PMTs), and simple timing electronics. This configuration allows to conduct fluorescence correlation spectroscopy (FCS) as well as smFRET of diffusing molecules. To comply with the spectral requirements of the PMT detectors in the setup, we introduce donor–acceptor pairs in the blue‐green spectral region (Alexa488, ATTO542, and Cy3B), for which we optimized the photon output with ferrocene‐based photostabilizers.

Despite all cutbacks and compromises made on the technical specifications of the FRET‐Brick and the selected dye combinations, we demonstrate that the setup is suitable for the relevant mainstream biological applications of smFRET and FCS with diffusing molecules. The device allows to characterize conformational changes in a macromolecule via relative changes in FRET efficiency or differentiate distinct interdye distances via the use of uncorrected apparatus‐dependent apparent FRET efficiencies E*. To maximize the usage of all information contained in our smFRET data, which lacks the advanced capabilities of MFD [16], PIE [13], or ALEX [18], we tested readily available parameters to construct multidimensional FRET histograms: the macrotime difference between donor and acceptor photons within a burst (Δ*T*_DA_) and the donor and acceptor brightness. We found that these parameters, particularly a 2D histogram of Δ*T*_DA_–E*, enable a straightforward identification of distinct FRET species. Using this setup and streamlined analysis approach, we demonstrate our capability of characterizing static oligonucleotide DNA standards with varying donor–acceptor distances and probing biologically relevant conformational changes in protein systems. Finally, we explored whether data from our setup can be used to obtain accurate FRET efficiency values to obtain real‐space interdye distances for oligonucleotide standards.

## 2 Results and Discussion

### 2.1 Design of the FRET‐Brick

**FIGURE 1:**
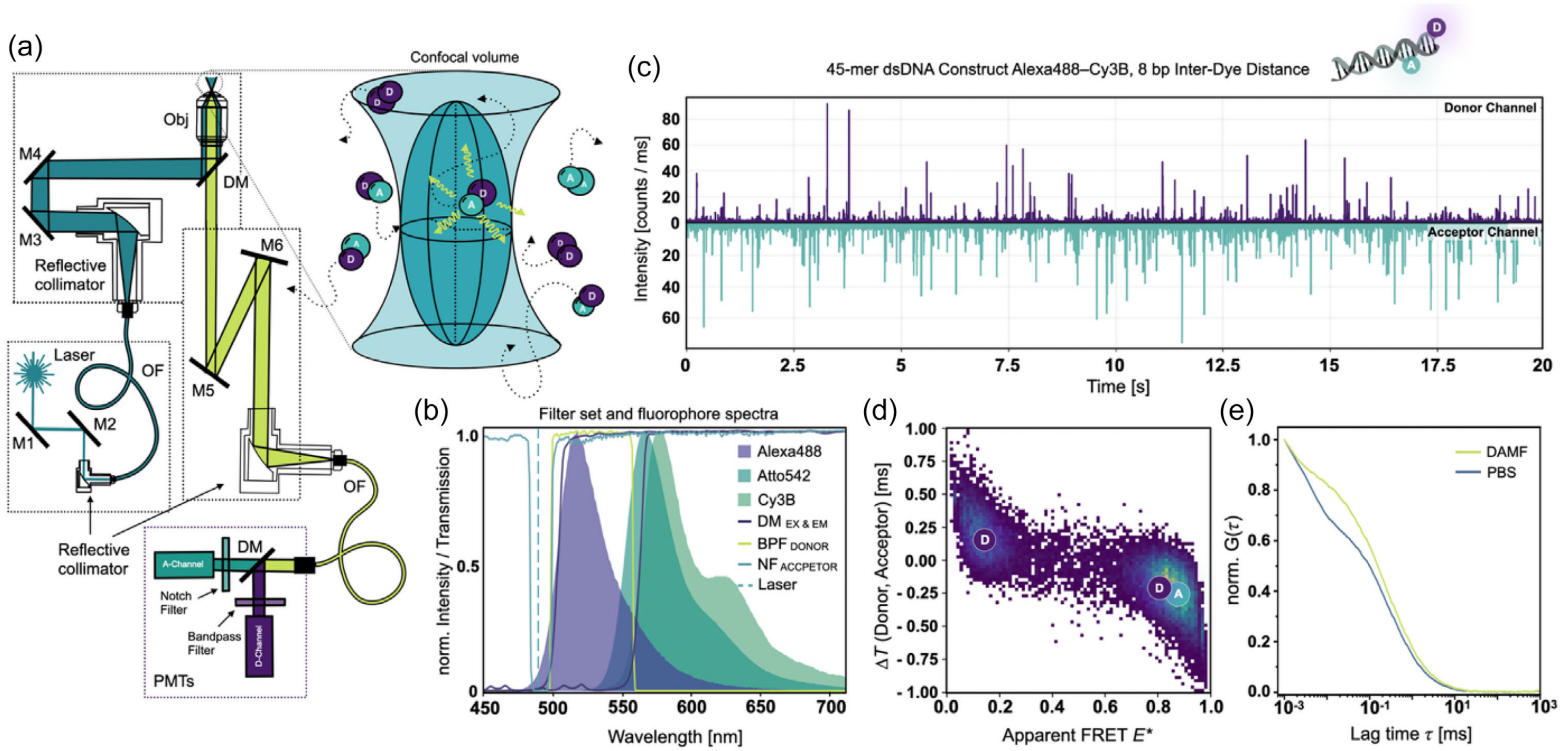
Overview of the FRET‐Brick. (a) Optical configuration of the FRET‐Brick. A 488 nm laser diode (Laser, blue) is coupled into an optical fiber (OF) and directed through the excitation layer onto the objective (Obj) to generate a confocal excitation volume. Freely diffusing, doubly labeled molecules carrying donor (D) and acceptor (A) fluorophores emit fluorescence bursts (light green) upon entering this volume. The emitted light is collected by the same objective, coupled into an OF, and delivered to a detection module (DM) where donor and acceptor signals separated by a dichroic mirror (DM) are detected by PMTs. (b) Spectral configuration. Excitation and emission dichroic mirrors (DC) are shown in purple, the bandpass filter (BPF) for the donor channel in light green, and the notch filter (NF) for the acceptor channel in blue. The dashed blue line shows the laser excitation wavelength. Emission spectra of available fluorophores—Alexa 488 (purple), Atto 542, and Cy3B (shades of green)—are overlaid to illustrate spectral overlap with the filter set and detector sensitivity. (c) Representative fluorescence time trace. Twenty‐second single‐molecule trace of a 100 pM dsDNA sample labeled with Alexa 488 (Donor channel) and Cy3B (Acceptor channel) at 8 bp interdye separation, recorded simultaneously in donor and acceptor channels under 488 nm excitation at 200 µW with 100 µM DAMF ((dimethylaminomethyl)ferrocene). (d) ΔT–E* histogram (apparent FRET efficiency, E*) versus macrotime difference between donor and acceptor photons, ΔT (Donor, Acceptor)) of the dsDNA sample shown in (c), where D denotes the donor‐only population and DA the FRET population. (e) FCS raw data of a dsDNA sample labeled with Alexa 488, excited at 15 kW cm^−2^ in PBS buffer with and without 100 µM DAMF.

With the main goal to reduce both the costs and the complexity of confocal single‐molecule detection, we chose the design of our previously published µFCS setup as a starting point for the FRET‐Brick. In the developed instrument, which is described and shown in full detail in Supplementary Note 1, cw excitation is supplied by a USB‐powered blue laser diode (488‐30‐1235‐BL, Q‐LINE) and combined with detection of fluorescence from diffusing single molecules by PMTs rather than avalanche photodiodes (APDs); Figure 1. As previously described [29], confocality is achieved by coupling of the emission beam into a single‐mode optical fiber (OF) with a diameter of 10 µm instead of a pinhole. This simplifies the overall optical design and allows autocalibration via two piezo mirrors M5/M6 (Figure 1a). The emission is then guided to an external detection box, where it is spectrally split before reaching the detectors. This configuration is advantageous because the system functions as a lens‐free microscope, apart from the objective.

Dichroic mirrors and emission filters were selected to match the new excitation (excitation dichroic ZT491rdc, Chroma) and emission wavelengths (emission dichroic ZT543rdc, Chroma), which are in line with the higher detection efficiency of the PMTs in the blue/green spectral range (Figure S1). To maximize the photon collection efficiency for both PMTs, a bandpass filter (BPF; FF03‐525/50, Semrock) and a notch filter (NF; NF488−15, Thorlabs) were used in the donor and acceptor channel, respectively. The latter filter blocks only reflected or scattered excitation light and and allows to collect the full acceptor emission spectrum (Figure 1b). This is particularly advantageous because the count sensitivity of PMTs decreases strongly toward longer wavelengths, dropping from about 2 × 10^5^ s^−1^ pW^−1^ at 550 nm to 1 × 10^5^ s^−1^ pW^−1^ at 600 nm and ≈5 × 10^3^ s^−1^ pW^−1^ near 700 nm (Figure S1). Data were collected using a USB counter module (USB‐CTR04, Measurement Computing), as introduced by the Gambin lab [30] and previously implemented in our FCS modality [29, 30], along with bespoke Python‐based acquisition software capable of registering data at 1 µs time resolution in the widely accepted photon hdf5 format [37].

Based on the optical configuration shown in Figure 1, we performed smFRET experiments with excitation at 488 nm at excitation intensities of 50–200 µW using a high‐NA water immersion objective (60×, NA 1.2, UPlanSAPO 60XW, Olympus, Japan). Epi‐fluorescence detection gave rise to count rates of both donor and acceptor dyes in the range of 10–100 kHz (Figure 1c). Thus, single‐molecule transits of double‐stranded 45‐mer oligonucleotide structures through the confocal volume were clearly visible as burst events (Figure 1c). For the isolation of burst data from our hdf5 data sets we used a customized version of ChiSurf [38], which are available as Jupyter Notebooks with this manuscript (see Data Availability statement). The software allows to obtain 1D and 2D histograms of apparent FRET efficiency, in which we found FRET populations to be clearly distinguished from single‐donor species (Figure 1d). Similarly, the data can be used for an autocorrelation analysis which provides the typical output of an FCS experiment, that is, diffusion and bunching times with a temporal resolution down to ∼1 µs, only limited by the time resolution of the used counter module (Figure 1e).

To evaluate the influence of the objective on detection performance, we compared several alternative objectives using FCS. The performance was assessed via the count rate per molecule (CPM) relative to the reference 60× NA 1.2 water immersion objective (UPlanSAPO 60XW, Olympus). A 60× NA 1.35 oil immersion objective (Olympus) showed a comparable CPM (∼104% relative to the reference), as expected for a similarly high numerical aperture. In contrast, a generic low‐cost OEM 100× NA 1.2 oil immersion objective and a generic OEM 60× NA 0.65 air objective exhibited substantially reduced CPM values of ∼17% and ∼1% of the reference objective, respectively (Figure S2). We further evaluated the suitability of these objectives for smFRET measurements. Representative smFRET time traces and apparent FRET efficiency histograms obtained with the three high‐NA immersion objectives are shown in Figure S3. All three immersion objectives allowed detection of single‐molecule burst events and determination of FRET efficiencies, with the expected reduction in photon count rates for the lower‐performing objectives. Thus, smFRET measurements remain technically feasible with these objectives, albeit requiring longer acquisition times to obtain comparable statistics. In contrast, the low‐NA air objective did not yield detectable burst events under the tested conditions and was therefore not suitable for smFRET measurements.

### 2.2 Optimization of Dye Combinations for FRET in the Blue‐Green Spectral Region

**FIGURE 2:**
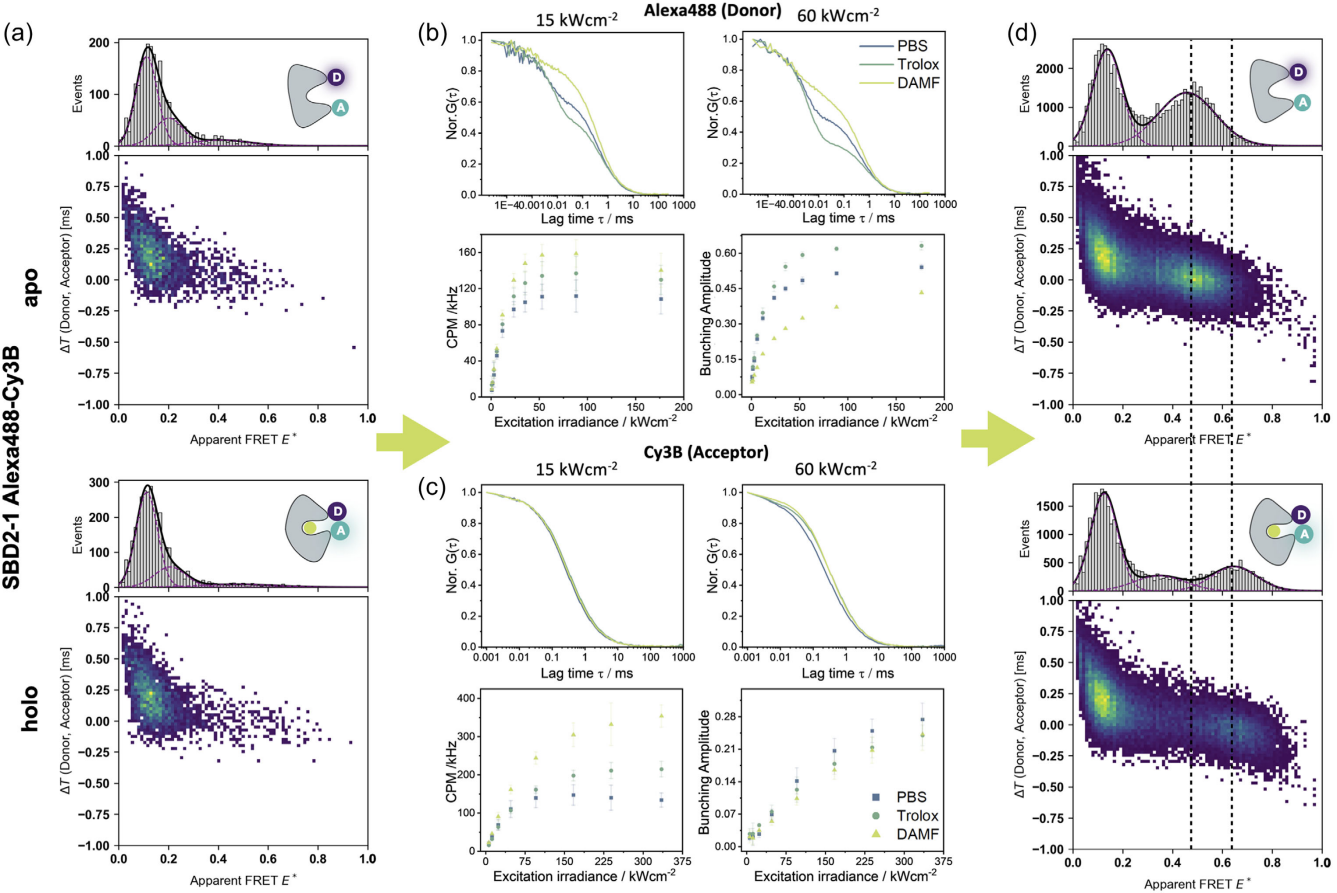
FCS‐guided assay optimization and photostabilizer screening. (a) Initial conditions of SBD2 in PBS buffer. Δ*T*–E^*^ histograms of SBD2 from *Lactococcus lactis* labeled with Alexa 488 (donor) and Cy3B (acceptor), recorded at 60 kW/cm^2^ laser excitation without photostabilizing additives. The upper panel shows the open (apo) conformation, and the lower panel shows the closed (holo) conformation in the presence of 1 mM glutamine. Apart from the donor‐only species, FRET‐active populations are barely visible due to rapid acceptor photobleaching under these conditions. (b,c) Photostabilizer screening via FCS. FCS of Alexa 488‐labeled dsDNA (b) and Cy3B‐labeled dsDNA; data for ATTO542 is presented in Figure S6 (c). Upper panels show representative normalized FCS curves at 15 kW/cm^2^ (left), and 60 kWcm^2^ (right) excitation power: blue, without additives; green, with 1 mM Trolox ((±)‐6‐hydroxy‐2,5,7,8‐tetramethylchromane‐2‐carboxylic acid); and yellow, with 100 µM DAMF ((dimethylaminomethyl)ferrocene). Lower panels summarize the power‐dependent screening results for molecular brightness (left) and triplet‐state amplitude (right), demonstrating the photostabilizing effects of Trolox and DAMF. (d) Optimized conditions of SBD2 in PBS with photostabilizers. Δ*T*–E^*^ histograms of the same SBD2−1 sample shown in (a), recorded under optimized conditions (30 kW/cm^2^ excitation, 100 µM DAMF, and 100 µM Trolox). Under these conditions, FRET‐active populations are clearly visible alongside donor‐only species, with the apo (open) conformation showing an apparent FRET efficiency of 0.46 and the holo (ligand‐bound, closed) conformation showing E^*^ = 0.65.

Blue and green fluorescent dyes that can serve as donor and acceptor pairs in FRET experiments have a small spectral separation and a limited photostability [39, 40, 41]. This poses challenges on the microscope design and the selection of matching fluorescent dyes. Based on our filter set and the dyes available in our stocks, for example, for protein or oligonucleotide labeling, the best candidates for FRET‐donors were Alexa Fluor 488 (Alexa488) and ATTO488 in combination with either Alexa555, ATTO542, or Cy3B as acceptor dyes. Each dye was characterized individually by using a 45‐mer double‐stranded DNA (dsDNA) sample via FCS measurements. With this, we tried to identify the condition that maximized molecular brightness before the onset of photobleaching in excitation power series. We balanced molecular brightness with photo‐induced bunching amplitudes observed in FCS, for instance, caused by formation triplet‐ or dark‐states. Based on our initial experiments, we discarded two dyes from a detailed screening process. Alexa488 was notably brighter compared to ATTO488 (Figure S4, Table S1) despite the structural similarity of the core dye [40, 41] (Figure S4a). On average and independently of the conditions, Alexa488 was more than twofold brighter than ATTO488 (Figure S4b, Table S1). Consequently, all FRET experiments were conducted with Alexa488 as donor dye. Alexa555 was also discarded from detailed analysis, since it showed much lower apparent FRET‐efficiency values compared to ATTO542‐ and Cy3B‐labeled samples both on DNA and proteins (Figure S5) despite a reasonable burst‐brightness in our experiments. For the remaining three dyes, we systematically optimized combinations of donor and acceptor dye pairs, laser excitation powers, and buffer additives for improved photostability. In that regard, we found it to be essential for high‐quality experiments in the FRET‐Brick to suppress dark‐state formation of the donor Alexa488 and to minimize acceptor photobleaching (Figure 2).

Alexa488 displayed a substantial bunching amplitude below 100 µs in PBS buffer; even at low excitation powers: >20% at 15 kW/cm^2^, ∼55% at 60 kW/cm^2^ (Figure 2b). In a FRET pair, the formation of triplet‐ or dark‐states of the donor dye will reduce the count rate and increase the shot‐noise of both, the donor and acceptor signal. Moreover, accurate FRET efficiencies require dark‐state corrected fluorescence quantum yields [1, 2]. On the other hand, bleaching of the acceptor within a burst results in a bridge between the donor–acceptor and donor‐only population, which complicates the determination of FRET efficiencies. To mitigate these effects, we first tested the gold‐standard for photostabilization Trolox [42] ((±)‐6‐Hydroxy‐2,5,7,8‐tetramethylchromane‐2‐carboxylic acid) [43]. Trolox showed a noticeable improvement of the Alexa488 count rate (expressed in counts per molecule, CPM). We note here that the CPM values reported in the FCS experiments in Figure 2 and Figure S6 were recorded on a Microtime 200 equipped with APD detectors. This implies that the reported CPM values are not the same as from the FRET‐Brick which uses PMT detection (shown in Figure 1e and Figure S4). To allow a comparison of both setups we conducted comparative FCS experiment on both setups which revealed an approximate 5‐10‐fold brightness difference attributed to the sensitivity difference of APDs and PMTs (Table S1 vs. Figure S6). Nevertheless, Trolox surprisingly increased the percentage of dark‐state formation, particularly in the desired high excitation intensity regime >15 kW/cm^2^ (Figure 2b). In search for alternative photostabilizers, we tested ferrocene derivatives [44], despite their ability for singlet‐quenching of fluorescein [45]. (Dimethylaminomethyl)ferrocene, abbreviated DAMF, proved to be an effective additive for Alexa488. The addition of 100 µM DAMF reduced the bunching amplitude from ≈55% to 30%. Our interpretation is that photoinduced electron transfer (PET [46]) occurs between the triplet‐state of Alexa488 and DAMF resulting in the formation of a radical anion from which the singlet ground‐state can be recovered by molecular oxygen. This ROXS‐process [47] increased the molecular brightness of Alexa 488 by ∼1.5‐fold to maximum values of 160 kHz at excitation powers >60 kW/cm^2^.

Strikingly, the effects of DAMF on the performance of Cy3B (Figure 2c) and ATTO542 (Figure S6) were similar and again DAMF outperformed Trolox. DAMF showed 1.5‐ to 3‐fold increases in CPM with a similar decrease in bunching amplitudes (Figure 2c). We were surprised about the competitive performance of DAMF and will present further results of similar stabilizers in upcoming work. Based on the optimized conditions for excitation intensity and photostabilizer conditions (100 µM DAMF), we performed FRET experiments with both combinations of donor and acceptor dyes on substrate‐binding domain 2 (SBD2), the soluble extracellular component of an amino‐acid import system of *Lactococcus lactis* [48]. Without any detailed analysis, the differences of the histogram quality between PBS buffer with (Figure 2d) and without the photostabilizer DAMF (Figure 2a) are strikingly clear and support the relevance of our screening efforts for optimized photostability.

### 2.3 smFRET Measurements with the FRET‐Brick

**FIGURE 3:**
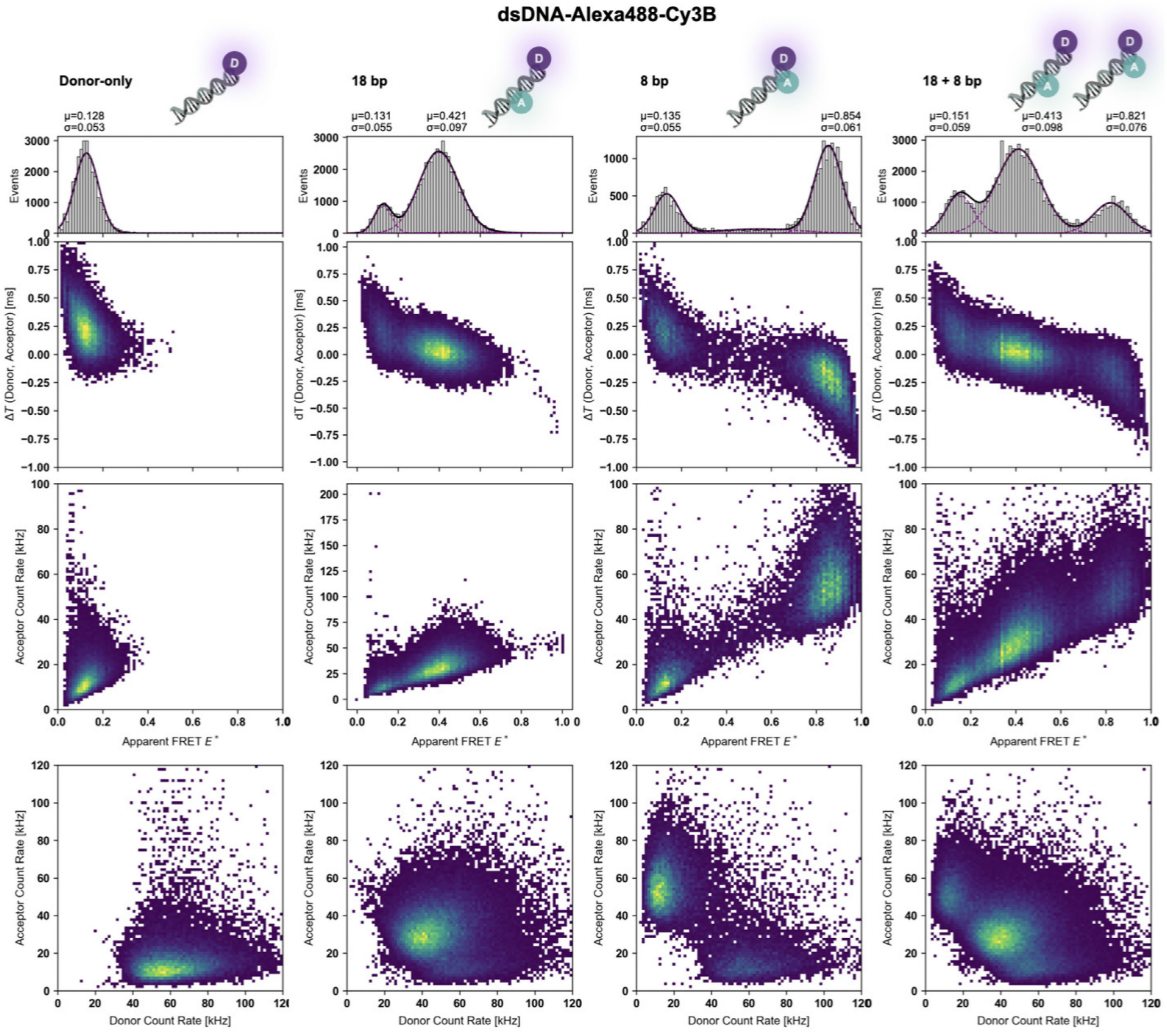
Multiparameter 2D histograms of Alexa 488–Cy3B labeled dsDNA samples. Two‐dimensional histograms of a 45‐mer dsDNA labeled with Alexa 488 (donor) and Cy3B (acceptor) at 60 kW/cm^2^ excitation in the presence of 100 µM DAMF: donor‐only, donor–acceptor dsDNA with 18 bp, 8 bp interdye distances, and a mix of the two. Rows correspond to different parameter spaces: (top) apparent FRET efficiency versus ΔT_DA_, (middle) apparent FRET efficiency versus acceptor brightness, and (bottom) acceptor versus donor brightness.

Using the optimized conditions, we performed FRET measurements on a series of double‐stranded DNA (dsDNA) and SBD2 protein samples to evaluate the performance and dynamic range of the FRET‐Brick. Initial reference measurements were carried out with 45‐mer dsDNA constructs [49, 50] with the dye pair Alexa488‐Cy3B. The experiments included donor‐only, acceptor‐only, and doubly labeled dsDNA with distinct interdye distance of 8 and 18 bp, as well as a mixed sample containing both 8 and 18 bp oligonucleotides (Figure 3 and Figure S7). The data were analyzed by initial burst identification and burst‐wise extraction of different parameters. These were burst‐wise proximity ratios to determine apparent FRET efficiencies E*, the macrotime difference ΔT_DA_ between donor and acceptor photons within a burst and donor and acceptor count rates.

The ΔT_DA_ represents the macrotime difference between donor and acceptor photons within a burst, calculated from photon counts binned in 1 µs intervals. Because each bin can contain multiple photons from both donor and acceptor channels, ΔT_DA_ does not reflect exact photon arrival times. Instead, it provides a coarse‐grained, statistical measure of the relative distribution of donor and acceptor photons within the burst. Values near zero indicate that donor and acceptor photons frequently appear within the same bins, positive values are related to bins dominated by donor photons, and negative values are bins dominated by acceptor photons. Donor and acceptor brightness reflect the total photon counts per molecule in each channel and vary due to FRET efficiency in the different samples.

From these parameters, we generated a series of 2D histograms to visualize population distributions with the goal of separating FRET‐active molecules from donor‐only species. The representations included apparent FRET efficiency E* versus ΔT_DA_ (Δ*T*–E* histogram), E* versus acceptor brightness (A–E* histogram), and donor versus acceptor count rate (D–A histogram); see different rows of Figure 3. In the donor‐only sample, the apparent FRET efficiency was 0.13, consistent with the expected donor leakage (lk). Overall, the donor‐only population was clearly distinguishable in all three plots. The acceptor‐only sample did not yield a measurable signal corresponding to the acceptor dye (Figure S7). Facilitated by the 2D plots, we observed that dsDNA with both donor and acceptor dye are readily distinguishable from donor‐only species. The 18 bp and 8 bp samples had E* values of 0.42 and 0.85, respectively, giving rise to a large dynamic distance range considering the Δbp of 10. Consequently, even subtle changes in interdye separation are clearly resolved. In the mixed 8 bp/18 bp sample, all three populations—donor‐only, 18 bp, and 8 bp—were resolved across all plots, further demonstrating the power of the multiparameter analysis for distinguishing coexisting species.

**FIGURE 4:**
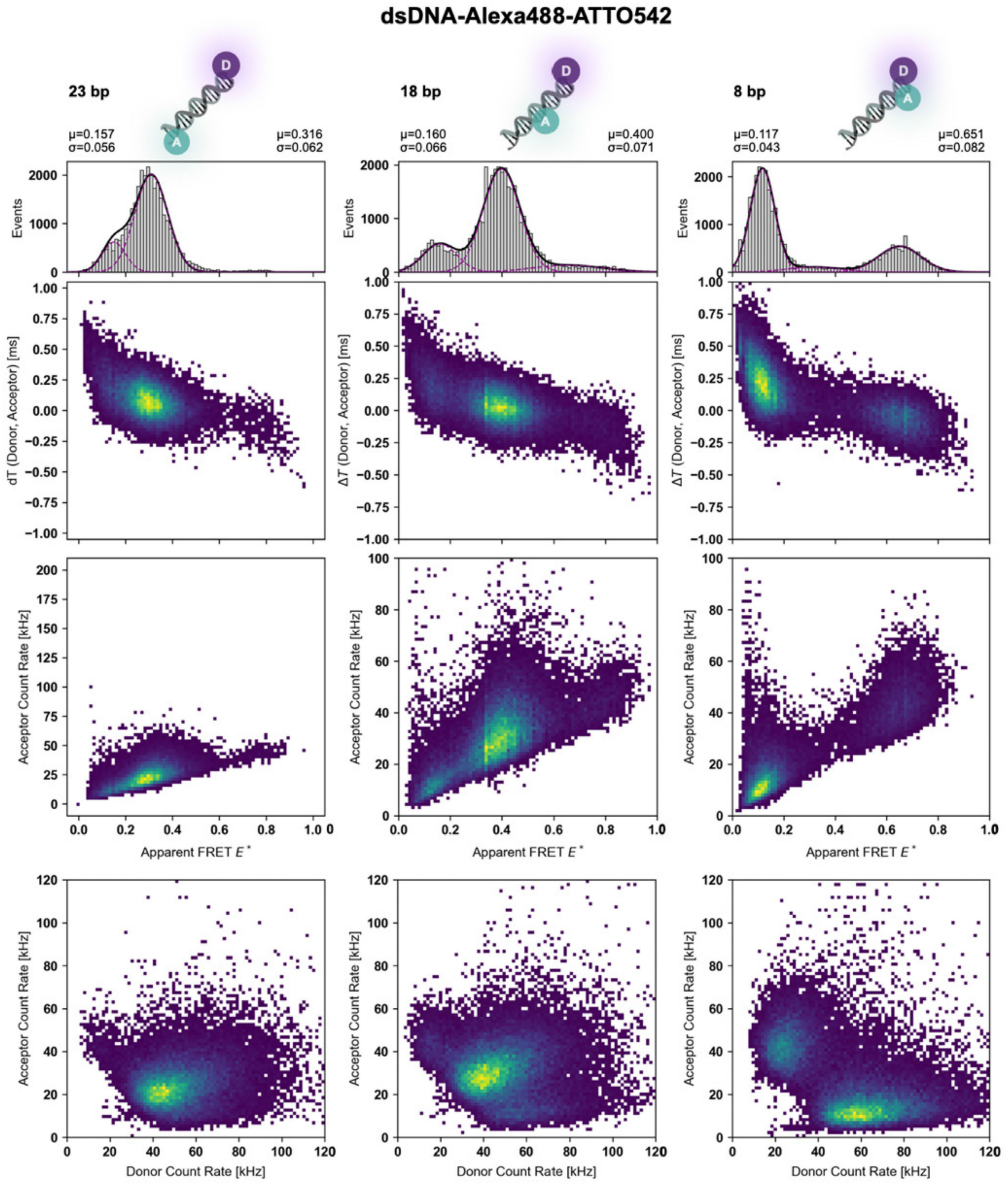
Multiparameter 2D histograms of Alexa488–ATTO542 labeled dsDNA samples. Two‐dimensional histograms of a 45‐mer dsDNA labeled with Alexa488 (donor) and ATTO542 (acceptor) at 60 kW/cm^2^ excitation in the presence of 100 µM DAMF. In each dataset for different donor–acceptor interdye distances, donor‐only species are clearly visible. Rows show different parameter spaces: (top) apparent FRET efficiency versus ΔT_DA_, (middle) apparent FRET efficiency versus acceptor brightness, and (bottom) acceptor versus donor brightness.

To assess the system's performance with a different dye pair, we studied the same dsDNA constructs with ATTO542 as acceptor dye (Figure 4) with interdye separations of 23 bp, 18 bp, and 8 bp. All samples had varying E* values according to their interdye separation and contained a clearly visible donor‐only species, which was straightforward to isolate in the 8 bp case. The apparent FRET efficiencies E* for this dye pair were 0.32 for the 23 bp construct, 0.40 for the 18 bp construct, and 0.65 for the 8 bp construct giving rise to a smaller dynamic range, either due to a different R_0_ value or enhanced photobleaching of ATTO542 compared to Cy3B. It was notable that some data sets (18 bp, 23 bp) contained subtle high‐FRET subpopulations, which have also been reported with this dsDNA construct with other fluorophores before [50], but might also be associated with small contamination of the sample with the 8 bp construct. In our view, this highlights the sensitivity of our analysis for detecting even minor coexisting species. Despite these minor contaminants, all main FRET‐active populations are clearly resolved, demonstrating that the system can reliably distinguish multiple constructs and effectively separate donor‐only and FRET‐active species across dye pairs with varying dynamic ranges.

**FIGURE 5:**
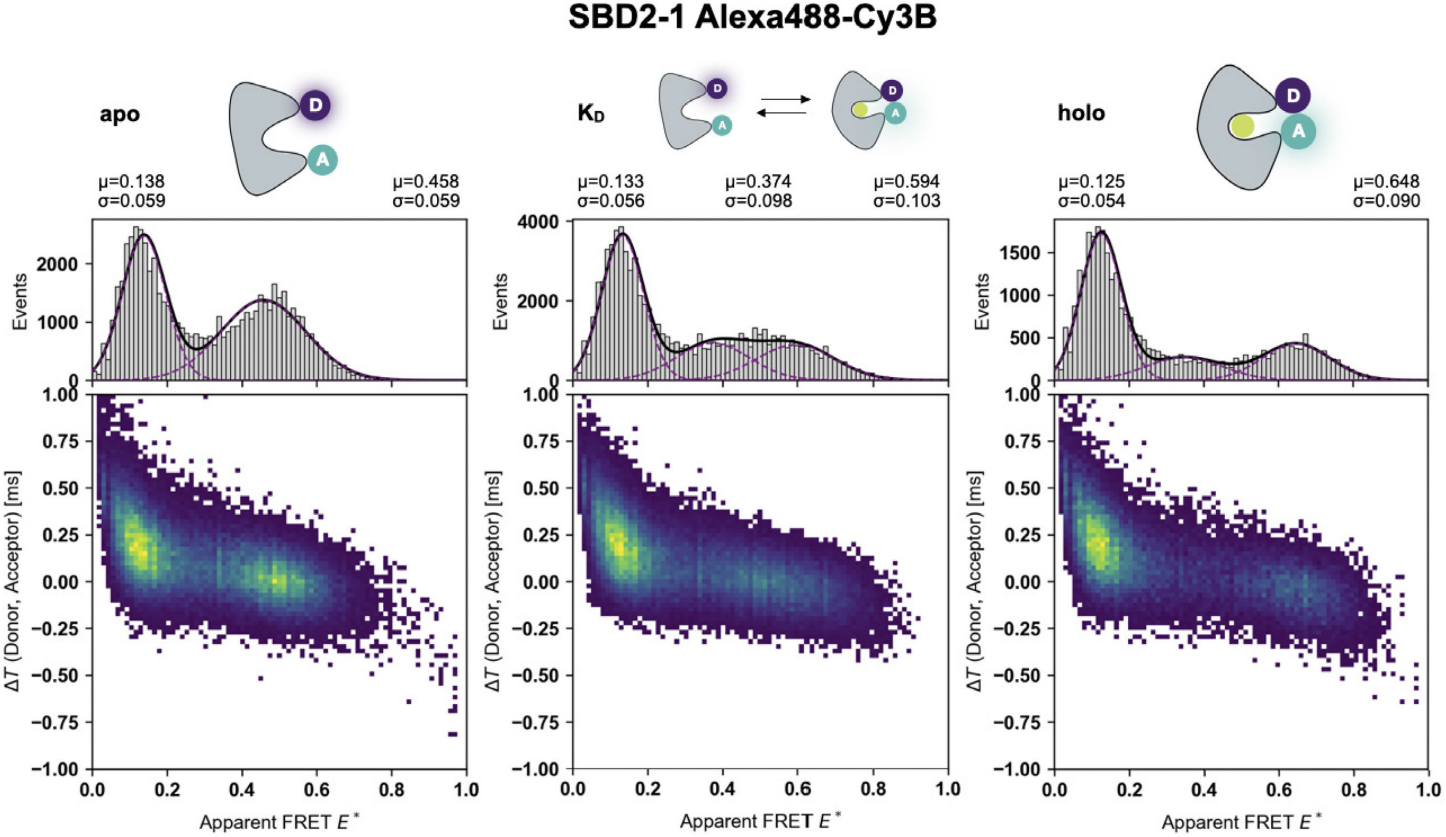
Multiparameter 2D histograms of SBD2 in different ligand‐bound states with photostabilizer DAMF and Trolox. Δ*T*–E^*^ histograms of SBD2 with Alexa 488 (donor) and Cy3B (acceptor) at 60 kW/cm^2^ excitation with 100 µM DAMF and 100 µM Trolox. The left panel shows the apo (open), the middle and right panels show the K_d_ (2 µM glutamine) conditions and holo state (1 mM glutamine), respectively.

Finally, to demonstrate the technical abilities of the FRET‐Brick beyond static DNA samples, FRET measurements were performed on SBD2 [48]. The protein adopts the well‐established periplasmic binding protein fold [51, 52] in which proteins undergo large conformational change upon ligand binding, transitioning from an open (apo) to a closed (holo) state [26, 28]. For the smFRET experiments, we used a published mutant in which the labeling sites (369C/451C) [27, 53] were positioned such that the FRET efficiency increases when the protein adopts the holo conformation upon ligand binding (Figure 5). Initial experiments with Alexa 488 and Cy3B on SBD2 showed elevated acceptor photobleaching even with DAMF added, which was not observed to the same extent in DNA samples. This is evident in the histograms as a bridge population connecting the donor‐only population and the donor–acceptor species (Figure S8). To mitigate this effect, the excitation power was reduced to 30 kW/cm^2^ and 100 µM Trolox was added additionally to stabilize the acceptor Cy3B (Figure 5 and Figure S9). Despite a notable improvement, a clear bridge population persisted (compare data in Figure S8 and S9).

It is worth noting that Δ*T*–E*, A–E*, and A–D histograms revealed FRET species that were well‐separated from the donor‐only peak at E* ∼0.14 (Figure 5, apo) and allowed a clear identification of photophysical processes. Based on the structure of SBD2 and previous publications, we clearly attribute the population at E* = 0.46 to the open conformation (E* = 0.46), which is dominant in the absence of ligand. Ligand addition induces a shift toward higher FRET efficiency (E* = 0.65) and represents the closed holo state of the protein adopted in the presence of glutamine. Both populations are observed at ligand conditions of 2 µM glutamine reflecting equal occupancy of the two conformational states. While this supports the idea of a functional protein, we observed that photobleaching was stronger in the holo state reducing the overall holo‐populations relative to the donor‐only (Figure 5). Under such conditions, determination of affinity values such as K_d_ via a titration will be systematically biased towards higher values, since more ligand is (apparently) needed to increase the fraction of the ligand‐bound holo state.

### 2.4 Benchmarking of Accurate FRET and Calculation of Interdye Distances

**FIGURE 6:**
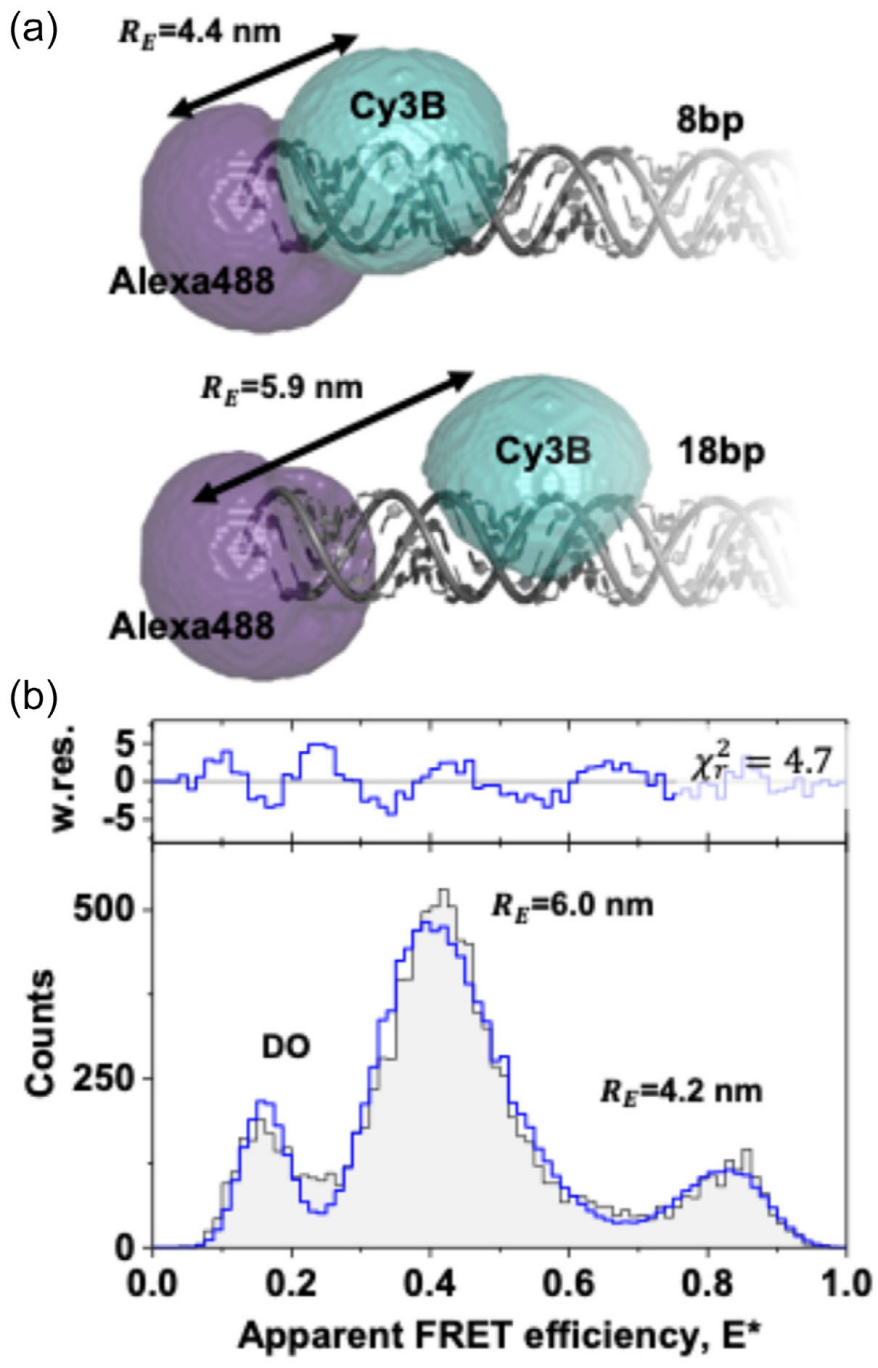
Simulated interdye distance and photon distribution analysis. (a) Accessible volume simulations for Alexa488 and Cy3B attached to dsDNA separated by 8 and 18 base pairs (bp). The distances, ‐$R_{E}$, correspond to FRET‐averaged distances. (b) Photon distribution analysis of a 8 bp and 18 bp Alexa488/Cy3B mixture. In the analysis, bursts were binned in 3 ms time‐windows (TWs), the photons recorded for the two detection channels were binned in a 2D histogram, which is marginalized to the apparent FRET efficiency, ‐$E_{app}$ (black line, gray area). The data was described by a mixture model with two normal distributions (SI Note 2, Equation 3) and a fraction of FRET‐inactive species (DO). The reported distances correspond the mean recovered distances. The spectral sensitives and excitation cross‐sections were determined by spectra, the emission cross talk of the donor in the acceptor channel was a free fitting parameter with a fit value of 0.156. To recover absolute distances, the manufacturer provided fluorescence quantum yield of Alexa488 (${\text{Φ}}_{F,D}=0.95$) and a reference value for Cy3B (${\text{Φ}}_{F,A}=0.85$) [55] were corrected by $b_{a}$, the power‐induced dark‐state population (${\text{Φ}}_{sm}=1-b_{a}{\text{Φ}}_{F}$). Absolute distances were determined using a spectrum‐derived [56] Förster radius ($R_{0}=6.5\;\mathrm{nm}$) and the dark‐state‐corrected quantum yields (${\text{Φ}}_{D,sm}\approx 0.8$, ${\text{Φ}}_{A,sm}\approx 0.65$).

As shown in the preceding sections, the FRET‐Brick and the established analysis procedure via Δ*T*–E* and brightness‐based histograms faithfully reproduce the Förster relation qualitatively for dsDNA and ligand‐induced conformational changes in SBD2. We finally wondered whether our approach and the information accessible are even sufficient to obtain accurate FRET efficiency values and interprobe distances. To benchmark our instrument, we use photon distribution analysis [54], a method that explicitly considers the photon shot‐noise, to maximize the attainable precision. We developed a model that accurately accounts for the excitation and emission cross talks (Supplementary Note 2, eqs. (3)–(11)), implemented the model in the open‐source software ChiSurf [38], and applied the model to dsDNA data to benchmark our instrument and test the ability to resolve species. The experimental histogram of the apparent FRET efficiencies E* of a mix of dsDNA with 8 bp/18 bp interdye distances of Alexa488/Cy3B was described by a Gaussian mixture model normally distributed in the interdye distances (Figure 6). In the analysis the mean interdye distances, the population fractions, the distribution width, and the fraction of FRET inactive species together with the spectral crosstalk from to donor to the acceptor channel were considered as variable parameters.

The spectral experimental correction parameters (see Figure 1b), that is, the spectral excitation and emission cross talks and the spectral sensitivities of the detectors for the dyes, were computed based on public (Cy3B) data [55, 56] or data provided by the manufacturer (ATTO542 and Alexa488). For Alexa488, the extinction coefficient at the used excitation wavelength of 488 nm was ∼75.000 L·mol^−1^·cm^−1^. At 488 nm, there is considerable direct excitation for ATTO542 and Cy3B considering the corresponding extinction coefficients of ∼13.000 L·mol^−1^·cm^−1^ and ∼28.600 L·mol^−1^·cm^−1^, suggesting excitation cross talks, $\alpha_{\mathrm{Exc}}=\sigma_{A|G}/\sigma_{D|G}$, of 0.22 (Alexa488/Atto542) and 0.48 (Alexa488/Cy3B). Based on the Alexa488 emission spectrum and the optical elements used in our setup, we anticipate a donor to acceptor crosstalk (fraction of donor emission detected in acceptor channel, α) of 0.096. Meanwhile, based on the spectra, we anticipate donor to acceptor crosstalk of 0.021 and 0.113 for Cy3B and ATTO542, respectively. This highlights the need to account for spectral crosstalk and sensitivities for accurate FRET analysis. Based on the spectral forms, the detection efficiency ratios of the FRET pair Alexa488/Cy3B and Alexa488/Atto543 were 2.21 and 2.29, respectively. Summing up, Alexa488/Cy3B has a lower emission crosstalk, yet a higher excitation crosstalk compared to Alexa488/ATTO542.

In the past, dsDNA served as standard in international round‐robin tests that showed an excellent agreement between FRET distanced modeled by accessible volumes (AVs) [8] and experiments [2]. Thus, we simulated the FRET‐averaged distances, $R_{E}$, between the labeling sites by using AV simulations (Figure 6a). The FRET distances based on AVs simulations with the experimentally recovered distances showed a good agreement (Figure 6b). Moreover, a comparison of the optimized emission cross talk (0.15) with the cross talk expected by the spectra (0.11) is consistent supporting the idea that the FRET‐Brick can be used for not only qualitative but also quantitative FRET studies.

## 3 Conclusion

While innovation in single‐molecule methods is often associated with increasing technical sophistication, that is, multicolor laser excitation [14, 57, 58], state‐of‐the‐art timing and detection electronics [13, 59, 60], complex optics [61] or automated sample handling [62, 63, 64], this progress also comes with higher cost and complexity, unintentionally widening the accessibility gap. Innovation, however, can also go into the opposite direction: reducing a system to its essential components to make it accessible to a broader range of users. This is particularly relevant as single‐molecule fluorescence approaches have become increasingly popular in biology and biochemistry.

Here, we demonstrate that smFRET can be performed with a minimal setup with auto‐alignment capabilities using a laser pointer for excitation, PMTs for detection, a minimal set of optics, and simple detection electronics. Rather than relying on multicolor excitation methods such as ALEX or PIE, we extracted meaningful information beyond the ratiometric FRET efficiency using basic burst‐parameters such as donor–acceptor count rates and macrotime differences between donor and acceptor photons. In the respective 2D histograms, we were able to identify FRET‐active molecules and to distinguish them from donor‐only species. With this, we were able to resolve distinct FRET efficiencies in static DNA constructs. With this, we obtained a good sense of the dynamic range of our instrument with different FRET pairs and were able to assess the conformational changes in a protein system. PDA and accurate FRET analysis even showed that the system was not even restricted to pure qualitative experiments of the FRET ruler (e.g., determination of relative distance changes), but that the data contained sufficient information to convert setup‐dependent E* values into interdye distances for oligonucleotide structures. Besides all technical aspects of our work, we also introduced a new compound for photostabilization of dye molecules in the blue‐green spectral region. Using the ferrocene‐based compound DAMF, we were able to significantly suppress dark‐state formation in both donor and acceptor dyes with a simultaneous increase of the fluorophore brightness. The use of this photostabilizer led to markedly better FRET histogram quality for both DNA and protein samples, and the success also motivated us to further study these molecules for other applications.

While we see room for improvement in both instrumentation and experimental conditions, this work serves as a proof of concept that reliable smFRET measurements can be achieved with a simple and robust optical setup. The PMTs used here were not optimized for the FRET‐assay but were simply available in the laboratory; detectors and optical layouts better suited for this spectral region could further enhance performance. Similarly, an optimization of the detection and the excitation volume outside of the diffraction‐limited regime and adjusting pinhole sizes [1], has also the potential to improve data quality. Finally, assay‐specific refinements, particularly for protein samples but also in the data analysis via open‐source code (ChiSurf), can further extend the applicability of our method.

Together, our instrument and the obtained results highlight that meaningful, quantitative single‐molecule insights can be obtained with an accessible, low‐cost apparatus. Simplifying the experimental framework while leveraging digital data processing offers a path toward making smFRET more broadly available across disciplines, bridging the gap between technical innovation and practical usability.

## Supporting Information

Additional supporting information can be found online in the Supporting Information section. Materials and Methods are provided in the Supplementary Information in Supplementary Note 1.

## Author Contributions

**Gabriel G.** **Moya Muñoz** and **Thorben Cordes** designed and conceived the study. **Gabriel G. Moya Muñoz**, **Jorge R. Luna** **Piedra** and **Thorben Cordes** built the setup. **Mostofa A. Rohman** and **Siyu Lu** provided data on photo‐stabilizers. **Gabriel G. Moya Muñoz**, **Jorge R. Luna Piedra** and **Pazit Con** conducted experiments. **Thomas‐Otavio Peulen** wrote data analysis software. **Gabriel G. Moya Muñoz**, **Pazit Con**, **Mostofa A. Rohman** and **Thomas‐Otavio Peulen** analyzed data. **Thorben Cordes** acquired funding and supervised the study. **Gabriel G. Moya Muñoz** prepared figures and wrote the initial draft of the manuscript together with **Thorben Cordes**. The manuscript was reviewed and approved by all authors.

## Additional Information

G.G.M. and T.C. declare commercial interest in Brick‐MIC as scientific co‐founders of FluoBrick Solutions GmbH, a company that distributes microspectroscopy setups.

## Supporting information

Supplementary Material

## Data Availability

The acquired experimental data and analysis software are accessible from Zenodo: 10.5281/zenodo.17921943.

## References

[1] G. Agam , C. Gebhardt , M. Popara , et al., “Reliability and Accuracy of Single‐Molecule FRET Studies for Characterization of Structural Dynamics and Distances in Proteins,” Nature Methods 20 (2023): 523–535. 36973549 10.1038/s41592-023-01807-0 PMC10089922

[2] B. Hellenkamp , S. Schmid , O. Doroshenko , et al., “Precision and Accuracy of Single‐Molecule FRET Measurements—a Multi‐Laboratory Benchmark Study,” Nature Methods 15 (2018): 669–676. 30171252 10.1038/s41592-018-0085-0 PMC6121742

[3] E. Sisamakis , A. Valeri , S. Kalinin , P. J. Rothwell , and C. A. M. Seidel , “Accurate Single‐Molecule FRET Studies Using Multi-parameter Fluorescence Detection,” Methods in Enzymology 475 (2010): 455–514. 20627168 10.1016/S0076-6879(10)75018-7

[4] E. Lerner , T. Cordes , A. Ingargiola , et al., “Toward Dynamic Structural Biology: Two Decades of Single‐Molecule Förster Resonance Energy Transfer,” Science 359 (2018): eaan1133. 29348210 10.1126/science.aan1133 PMC6200918

[5] E. Lerner , A. Barth , J. Hendrix , et al., “FRET‐Based Dynamic Structural Biology: Challenges, Perspectives and an Appeal for Open‐Science Practices,” ELife 10, e60416. 33779550 10.7554/eLife.60416 PMC8007216

[6] N. K. Lee , A. N. Kapanidis , Y. Wang , et al., “Accurate FRET Measurements Within Single Diffusing Biomolecules Using Alternating‐Laser Excitation,” Biophysical Journal 88 (2005): 2939–2953. 15653725 10.1529/biophysj.104.054114 PMC1282518

[7] A. Muschielok , J. Andrecka , A. Jawhari , F. Brückner , P. Cramer , and J. Michaelis , “A Nano‐Positioning System for Macromolecular Structural Analysis,” Nature Methods 5 (2008): 965–971. 18849988 10.1038/nmeth.1259

[8] S. Kalinin , T. Peulen , S. Sindbert , et al., “A Toolkit and Benchmark Study for FRET‐Restrained High‐Precision Structural Modeling,” Nature Methods 9 (2012): 1218–1225. 23142871 10.1038/nmeth.2222

[9] B. Hellenkamp , P. Wortmann , F. Kandzia , M. Zacharias , and T. Hugel , “Multidomain Structure and Correlated Dynamics Determined by Self‐Consistent FRET Networks,” Nature Methods 14 (2017): 174–180. 27918541 10.1038/nmeth.4081 PMC5289555

[10] T. D. Craggs and A. N. Kapanidis , “Six Steps Closer to FRET‐Driven Structural Biology,” Nature Methods 9 (2012): 1157–1158. 23223168 10.1038/nmeth.2257

[11] M. Dimura , T. O. Peulen , C. A. Hanke , A. Prakash , H. Gohlke , and C. A. Seidel , “Quantitative FRET Studies and Integrative Modeling Unravel the Structure and Dynamics of Biomolecular Systems,” Current Opinion in Structural Biology 40 (2016): 163–185. 27939973 10.1016/j.sbi.2016.11.012

[12] J. Hohlbein , T. D. Craggs , and T. Cordes , “Alternating‐Laser Excitation: Single‐Molecule FRET and beyond,” Chemical Society Reviews 43 (2014): 1156–1171. 24037326 10.1039/c3cs60233h

[13] B. K. Müller , E. Zaychikov , C. Bräuchle , and D. C. Lamb , “Pulsed Interleaved Excitation,” Biophysical Journal 89 (2005): 3508–3522. 16113120 10.1529/biophysj.105.064766 PMC1366845

[14] E. Bilgen and D. C. Lamb , “Multicolor Single‐Molecule FRET Studies on Dynamic Protein Systems,” Current Opinion in Structural Biology 93 (2025): 103117. 40664122 10.1016/j.sbi.2025.103117

[15] L. Bonhomme , E. Bilgen , C. Clerté , et al., “Triple Labeling Resolves a GPCR Intermediate State by Using Three‐Color Single Molecule FRET,” Journal of the American Chemical Society 147 (2025): 17689–17700. 40373293 10.1021/jacs.4c18364 PMC12123625

[16] J. Widengren , V. Kudryavtsev , M. Antonik , S. Berger , M. Gerken , and C. A. M. Seidel , “Single‐Molecule Detection and Identification of Multiple Species by Multiparameter Fluorescence Detection,” Analytical Chemistry 78 (2006): 2039–2050. 16536444 10.1021/ac0522759

[17] P. J. Rothwell , S. Berger , O. Kensch , et al., “Multiparameter Single‐Molecule Fluorescence Spectroscopy Reveals Heterogeneity of HIV-1 Reverse Transcriptase: Primer/Template Complexes,” Proceedings of the National Academy of Sciences 100 (2003): 1655–1660. 10.1073/pnas.0434003100 PMC149888 12578980

[18] A. N. Kapanidis , N. K. Lee , T. A. Laurence , S. Doose , E. Margeat , and S. Weiss , “Fluorescence‐Aided Molecule Sorting: Analysis of Structure and Interactions by Alternating‐Laser Excitation of Single Molecules,” Proceedings of the National Academy of Sciences of the United States of America 101 (2004): 8936–8941. 15175430 10.1073/pnas.0401690101 PMC428450

[19] A. N. Kapanidis , T. A. Laurence , N. K. Lee , E. Margeat , X. Kong , and S. Weiss , “Alternating‐Laser Excitation of Single Molecules,” Accounts of Chemical Research 38 (2005): 523–533. 16028886 10.1021/ar0401348

[20] F. Huang , S. Sato , T. D. Sharpe , L. Ying , and A. R. Fersht , “Distinguishing between Cooperative and Unimodal Downhill Protein Folding,” Proceedings of the National Academy of Sciences 104 (2007): 123–127. 10.1073/pnas.0609717104 PMC1765421 17200301

[21] F. Husada , K. Bountra , K. Tassis , et al., “Conformational Dynamics of the ABC Transporter McjD Seen by Single‐Molecule FRET,” The EMBO Journal 37 (2018): e100056. 30237313 10.15252/embj.2018100056 PMC6213277

[22] D. Scheerer , D. Levy , R. Casier , I. Riven , H. Mazal , and G. Haran , “Interplay between Conformational Dynamics and Substrate Binding Regulates Enzymatic Activity: A Single‐Molecule FRET Study,” Chemical Science 16 (2025): 3066–3077. 39877815 10.1039/d4sc06819j PMC11770808

[23] D. S. Majumdar , I. Smirnova , V. Kasho , et al., “Single‐Molecule FRET Reveals Sugar‐Induced Conformational Dynamics in LacY,” Proceedings of the National Academy of Sciences 104 (2007): 12640–12645. 10.1073/pnas.0700969104 PMC1937519 17502603

[24] J. Fort , A. Nicolàs‐Aragó , L. Maggi , et al., “The Conserved Lysine Residue in Transmembrane helix 5 Is Pivotal for the Cytoplasmic Gating of the L- Amino Acid Transporters,” PNAS Nexus 4 (2025): 584. 10.1093/pnasnexus/pgae584 PMC11736713 39822574

[25] Y. Santoso , J. P. Torella , and A. N. Kapanidis , “Characterizing Single‐Molecule FRET Dynamics with Probability Distribution Analysis,” Chemphyschem : A European Journal of Chemical Physics and Physical Chemistry 11 (2010): 2209–2219. 20575136 10.1002/cphc.201000129

[26] M. de Boer , G. Gouridis , R. Vietrov , et al., “Conformational and Dynamic Plasticity in Substrate‐Binding Proteins Underlies Selective Transport in ABC Importers,” ELife 8 (2019): e44652. 30900991 10.7554/eLife.44652 PMC6450668

[27] G. Gouridis , Y. A. Muthahari , M. De Boer , et al., “Structural Dynamics in the Evolution of a Bilobed Protein Scaffold,” Proceedings of the National Academy of Sciences 118 (2021): e2026165118. 10.1073/pnas.2026165118 PMC8694067 34845009

[28] E. Ploetz , G. K. Schuurman‐Wolters , N. Zijlstra , et al., “Structural and Biophysical Characterization of the Tandem Substrate‐Binding Domains of the ABC Importer GlnPQ,” Open Biology 11 (2021): 200406. 33823661 10.1098/rsob.200406 PMC8025302

[29] G. G. Moya Muñoz , O. Brix , P. Klocke , et al., “Single‐molecule detection and super‐resolution imaging with a portable and adaptable 3D‐printed microscopy platform (Brick‐MIC),” Science Advances 10 (2024): eado3427. 39321299 10.1126/sciadv.ado3427 PMC11423890

[30] J. W. P. Brown , A. Bauer , M. E. Polinkovsky , et al., “Single-Molecule Detection on a Portable 3D‐Printed Microscope,” Nature Communications 10 (2019): 5662. 10.1038/s41467-019-13617-0 PMC6906517 31827096

[31] R. Strack , “The miCube Open Microscope,” Nature Methods 16 (2019): 958. 10.1038/s41592-019-0607-4 31562480

[32] B. Ambrose , J. M. Baxter , J. Cully , et al., “The smfBox Is an Open‐Source Platform for Single‐Molecule FRET,” Nature Communications 11 (2020): 5641. 10.1038/s41467-020-19468-4 PMC7648814 33159061

[33] B. Diederich , R. Lachmann , S. Carlstedt , et al., “A Versatile and Customizable Low‐Cost 3D‐Printed Open Standard for Microscopic Imaging,” Nature Communications 11 (2020): 5979. 10.1038/s41467-020-19447-9 PMC7688980 33239615

[34] A. C. Zehrer , A. Martin‐Villalba , B. Diederich , and H. Ewers , “An Open‐Source, High Resolution, Automated Fluorescence Microscope,” (2024), 10.7554/eLife.89826.2. PMC10942636 38436658

[35] M. Loretan , M. Barella , N. Fuchs , et al., “Direct Single‐Molecule Detection and Super‐Resolution Imaging with a Low‐Cost Portable Smartphone‐Based Microscope,” Nature Communications 16 (2025): 8937. 10.1038/s41467-025-63993-z PMC12508040 41062474

[36] J. Lightley , S. Kumar , M. Q. Lim , et al., “OpenFrame : A Modular, Sustainable, Open Microscopy Platform with Single‐Shot, Dual‐Axis Optical Autofocus Module Providing High Precision and Long Range of Operation,” Journal of Microscopy 292 (2023): 64–77. 37616077 10.1111/jmi.13219 PMC10953376

[37] A. Ingargiola , T. Laurence , R. Boutelle , S. Weiss , and X. Michalet , “Photon‐HDF5: An Open File Format for Timestamp‐Based Single‐Molecule Fluorescence Experiments,” Biophysical Journal 110 (2016): 26–33. 26745406 10.1016/j.bpj.2015.11.013 PMC4805879

[38] T.-O. Peulen , O. Opanasyuk , and C. A. M. Seidel , “Combining Graphical and Analytical Methods with Molecular Simulations To Analyze Time‐Resolved FRET Measurements of Labeled Macromolecules Accurately,” The Journal of Physical Chemistry. B 121 (2017): 8211–8241. 28709377 10.1021/acs.jpcb.7b03441 PMC5592652

[39] H. Wallrabe , M. Stanley , A. Periasamy , and M. Barroso , “One- and Two‐Photon Fluorescence Resonance Energy Transfer Microscopy to Establish a Clustered Distribution of Receptor‐Ligand Complexes in Endocytic Membranes,” Journal of Biomedical Optics 8 (2003): 339. 12880337 10.1117/1.1584444

[40] A. Konrad , M. Metzger , A. M. Kern , M. Brecht , and A. J. Meixner , “Revealing the Radiative and Non‐Radiative Relaxation Rates of the Fluorescent Dye Atto488 in a λ/2 Fabry–Pérot‐Resonator by Spectral and Time Resolved Measurements,” Nanoscale 8 (2016): 14541–14547. 27414019 10.1039/c6nr02380k

[41] N. Panchuk‐Voloshina , R. P. Haugland , J. Bishop‐Stewart , et al., “Alexa Dyes, a Series of New Fluorescent Dyes that Yield Exceptionally Bright, Photostable Conjugates,” Journal of Histochemistry & Cytochemistry 47 (1999): 1179–1188. 10449539 10.1177/002215549904700910

[42] I. Rasnik , S. A. McKinney , and T. Ha , “Nonblinking and Long‐Lasting Single‐Molecule Fluorescence Imaging,” Nature Methods 3 (2006): 891–893. 17013382 10.1038/nmeth934

[43] T. Cordes , J. Vogelsang , and P. Tinnefeld , “On the Mechanism of Trolox as Antiblinking and Antibleaching Reagent,” Journal of the American Chemical Society 131 (2009): 5018–5019. 19301868 10.1021/ja809117z

[44] T. Cordes , I. H. Stein , C. Forthmann , et al., Tinnefeld in Advanced Microscopy Techniques (Munich: OSA, 2009). 7367_1D

[45] L. Zhang , C. Wang , Y. Li , et al., “Modular Design and Scaffold‐Synthesis of Multi‐Functional Fluorophores for Targeted Cellular Imaging and Pyroptosis,” Angewandte Chemie International Edition 64 (2025): e202415627. 39555698 10.1002/anie.202415627 PMC11753610

[46] H. Niu , J. Liu , H. M. O’Connor , T. Gunnlaugsson , T. D. James , and H. Zhang , “Photoinduced Electron Transfer (PeT) Based Fluorescent Probes for Cellular Imaging and Disease Therapy,” Chemical Society Reviews 52 (2023): 2322–2357. 36811891 10.1039/d1cs01097b

[47] J. Vogelsang , R. Kasper , C. Steinhauer , et al., “A Reducing and Oxidizing System Minimizes Photobleaching and Blinking of Fluorescent Dyes,” Angewandte Chemie International Edition 47 (2008): 5465–5469. 18601270 10.1002/anie.200801518

[48] G. Gouridis , G. K. Schuurman‐Wolters , E. Ploetz , et al., “Conformational Dynamics in Substrate‐Binding Domains Influences Transport in the ABC Importer GlnPQ,” Nature Structural & Molecular Biology 22 (2015): 57–64. 10.1038/nsmb.2929 25486304

[49] L. Le Reste , J. Hohlbein , K. Gryte , and A. N. Kapanidis , “Characterization of Dark Quencher Chromophores as Nonfluorescent Acceptors for Single‐Molecule FRET,” Biophysical Journal 102 (2012): 2658–2668. 22713582 10.1016/j.bpj.2012.04.028 PMC3368131

[50] E. Ploetz , E. Lerner , F. Husada , et al., “Förster Resonance Energy Transfer and Protein‐Induced Fluorescence Enhancement as Synergetic Multi‐Scale Molecular Rulers,” Scientific Reports 6 (2016): 33257. 27641327 10.1038/srep33257 PMC5027553

[51] R. P.-A. Berntsson , S. H. J. Smits , L. Schmitt , D.-J. Slotboom , and B. Poolman , “A Structural Classification of Substrate‐Binding Proteins,” FEBS Letters 584 (2010): 2606–2617. 20412802 10.1016/j.febslet.2010.04.043

[52] G. H. Scheepers , J. A. Lycklama A. Nijeholt , and B. Poolman , “An Updated Structural Classification of Substrate‐Binding Proteins,” FEBS Letters 590 (2016): 4393–4401. 27714801 10.1002/1873-3468.12445

[53] M. F. Peter , C. Gebhardt , R. Mächtel , et al., “Cross‐Validation of Distance Measurements in Proteins by PELDOR/DEER and Single‐Molecule FRET,” Nature Communications 13 (2022): 4396. 10.1038/s41467-022-31945-6 PMC9338047 35906222

[54] S. Kalinin , S. Felekyan , M. Antonik , and C. A. M. Seidel , “Probability Distribution Analysis of Single‐Molecule Fluorescence Anisotropy and Resonance Energy Transfer,” The Journal of Physical Chemistry. B 111 (2007): 10253–10262. 17676789 10.1021/jp072293p

[55] M. E. Sanborn , B. K. Connolly , K. Gurunathan , and M. Levitus , “Fluorescence Properties and Photophysics of the Sulfoindocyanine Cy3 Linked Covalently to DNA,” The Journal of Physical Chemistry. B 111 (2007): 11064–11074. 17718469 10.1021/jp072912u

[56] T. J. Lambert , “FPbase: A Community‐Editable Fluorescent Protein Database,” Nature Methods 16 (2019): 277–278. 30886412 10.1038/s41592-019-0352-8

[57] X. A. Feng , M. F. Poyton , and T. Ha , “Multicolor Single‐Molecule FRET for DNA and RNA Processes,” Current Opinion in Structural Biology 70 (2021): 26–33. 33894656 10.1016/j.sbi.2021.03.005 PMC8528908

[58] I. H. Stein , C. Steinhauer , and P. Tinnefeld , “Single‐Molecule Four‐Color FRET Visualizes Energy‐Transfer Paths on DNA Origami,” Journal of the American Chemical Society 133 (2011): 4193–4195. 21250689 10.1021/ja1105464

[59] V. Kudryavtsev , M. Sikor , S. Kalinin , D. Mokranjac , C. A. M. Seidel , and D. C. Lamb , “Combining MFD and PIE for Accurate Single‐Pair Förster Resonance Energy Transfer Measurements,” Chemphyschem : A European Journal of Chemical Physics and Physical Chemistry 13 (2012): 1060–1078. 22383292 10.1002/cphc.201100822

[60] T. A. Laurence , X. Kong , M. Jäger , and S. Weiss , “Probing Structural Heterogeneities and Fluctuations of Nucleic Acids and Denatured Proteins,” Proc. Natl. Acad. Sci. U.S.A 102 (2005): 17348–17353. 16287971 10.1073/pnas.0508584102 PMC1297681

[61] A. Ingargiola , R. A. Colyer , D. Kim , et al., California, USA 2012). 82280B.

[62] R. Kiselev , R. A. Brady , A. Modak , et al., “Parallel Stopped‐Flow Interrogation of Diverse Biological Systems at the Single‐Molecule Scale,” Nature Methods (2026), 10.1038/s41592-025-02944-4. PMC12791014 41331139

[63] A. Hartmann , K. Sreenivasa , M. Schenkel , et al., “An Automated Single‐Molecule FRET Platform for High‐Content, Multiwell Plate Screening of Biomolecular Conformations and Dynamics,” Nature Communications 14 (2023): 6511. 10.1038/s41467-023-42232-3 PMC10579363 37845199

[64] S. Kim , A. M. Streets , R. R. Lin , S. R. Quake , S. Weiss , and D. S. Majumdar , “High‐Throughput Single‐Molecule Optofluidic Analysis,” Nature Methods 8 (2011): 242–245. 21297618 10.1038/nmeth.1569 PMC3075913

