# Supplementary material for: Single‐Molecule Forster Resonance Energy Transfer With a Minimalistic 3D‐Printed Setup and Dyes in the Blue‐Green Spectral Region
Source: Chemphyschem. 2026 Apr 30;27(8):e202500909. doi: 10.1002/cphc.202500909 (PMC13131691; doi:10.1002/cphc.202500909)
Supplement: Supplementary file 1 — Supplementary Material [file CPHC-27-e202500909-s001.pdf]

Supplementary Information for the article

Single-molecule FRET with a minimalistic 3D-printed setup and dyes in the blue-green spectral region

Gabriel G. Moya Muñoz[1,2,3,#,+*], Jorge R. Luna Piedra[2,+], Pazit Con[2,3,+], Mostofa A. Rohman[1], Siyu Lu[1,2], Thomas-Otavio Peulen[1,*] and Thorben Cordes[1,2*]

[1] Biophysical Chemistry, Department of Chemistry and Chemical Biology, Technische Universität Dortmund, Otto-Hahn-Str. 4a, 44227 Dortmund, Germany

[2] Physical and Synthetic Biology, Faculty of Biology, Ludwig-Maximilians-Universität München, Großhadernerstr. 2-4, 82152 Planegg-Martinsried, Germany

[3] Department of Poultry and Aquaculture, Institute of Animal Sciences, Agricultural Research Organization, Volcani Center, Rishon LeZion, Israel

*corresponding authors:
moya@fluobrick.com, thomas.peulen@tu-dortmund.de, thorben.cordes@tu-dortmund.de

[+]these authors contributed equally to this work

[#]current affiliation: FluoBrick Solutions GmbH, Rudower Chaussee 29, 12489 Berlin, Germany

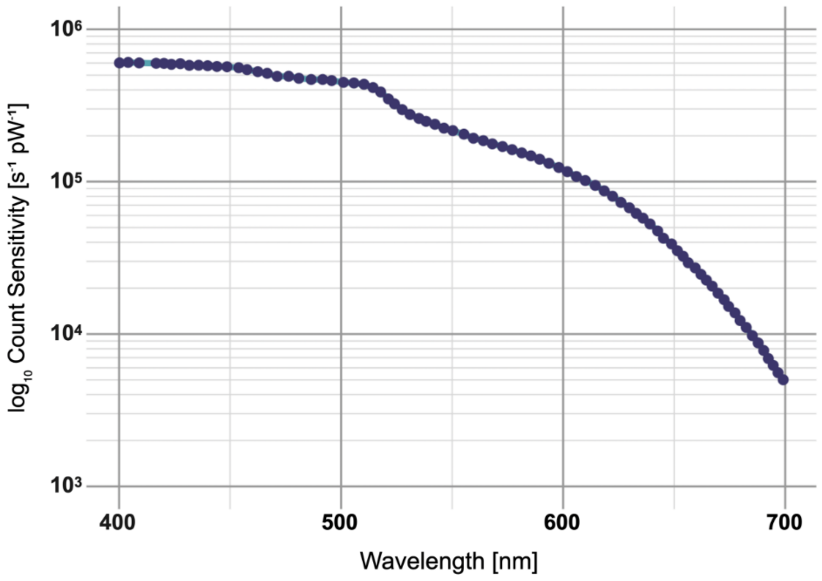

Figure S1: Wavelength-dependent photon count sensitivity of the Hamamatsu H10682-210 photomultiplier tube (PMT). Data extracted from the manufacturer's specifications.

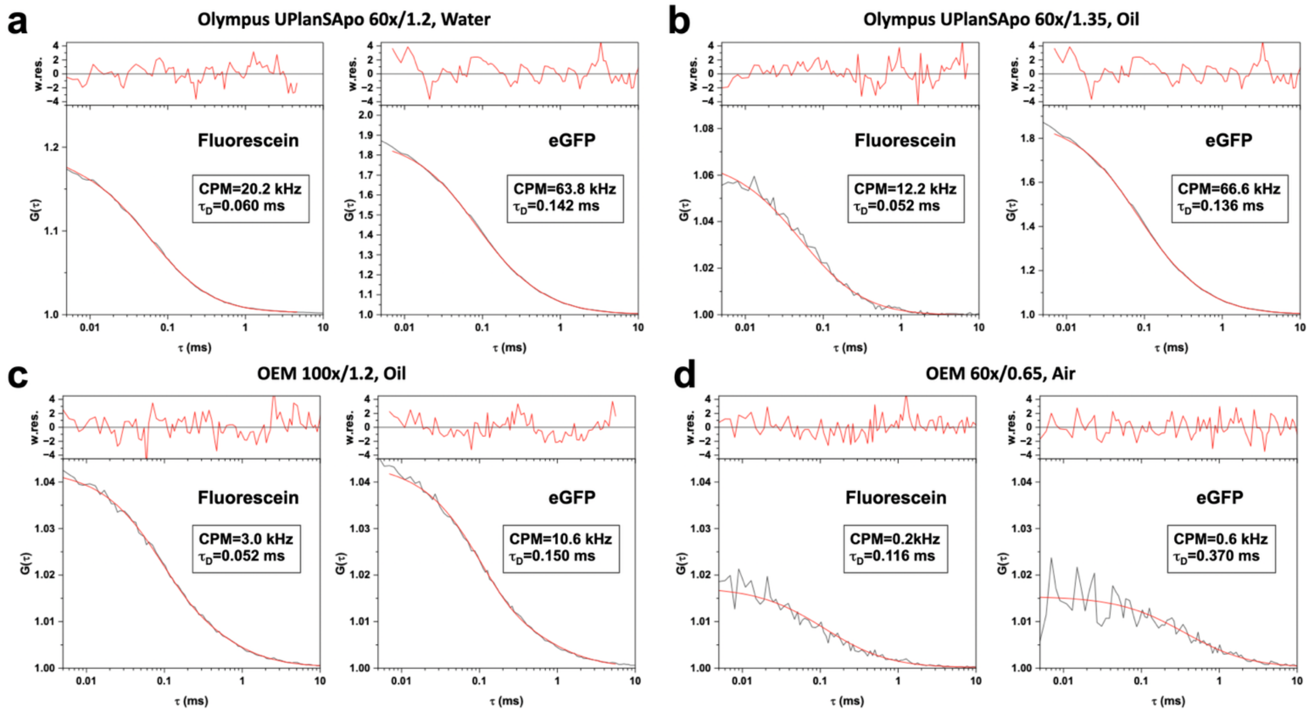

Figure S2: Objective-dependent fluorescence correlation spectroscopy (FCS) measurements. Autocorrelation curves recorded for fluorescein and eGFP using four different microscope objectives at an excitation power of 100 µW (measured after the objective). Panels correspond to (a) 60× NA 1.2 water immersion objective (Olympus), (b) 60× NA 1.3 oil immersion objective (Olympus), (c) generic OEM 100× NA 1.2 oil immersion objective, and (d) generic OEM 60× NA 0.65 air objective. Experimental data are shown as black curves, while the corresponding fits are shown in red.

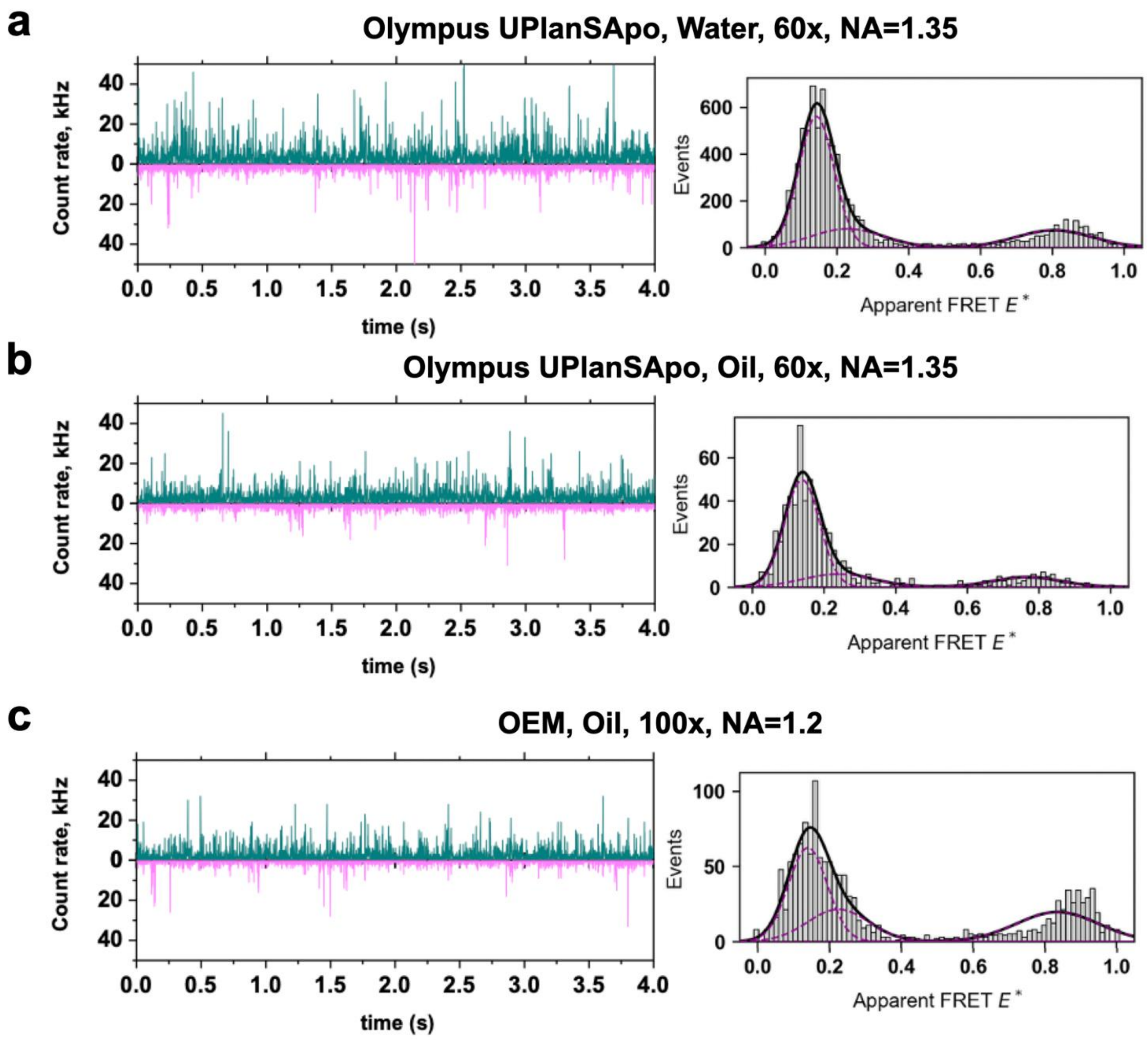

Figure S3: smFRET measurements obtained with different microscope objectives. Representative single-molecule time traces (left, 4 s duration) and corresponding apparent FRET efficiency histograms (right) recorded for a 45-mer dsDNA labeled with Alexa 488 (donor) and Cy3B (acceptor) with an 8 bp interdye separation. Measurements were performed using (a) a 60× NA 1.2 water immersion objective (Olympus), (b) a 60× NA 1.3 oil immersion objective (Olympus), and (c) a generic OEM 100× NA 1.2 oil immersion objective. All measurements were conducted at an excitation power of 100 µW (measured after the objective) in the presence of 100 µM DAMF. Apparent FRET efficiency histograms were obtained from 5 min measurements.

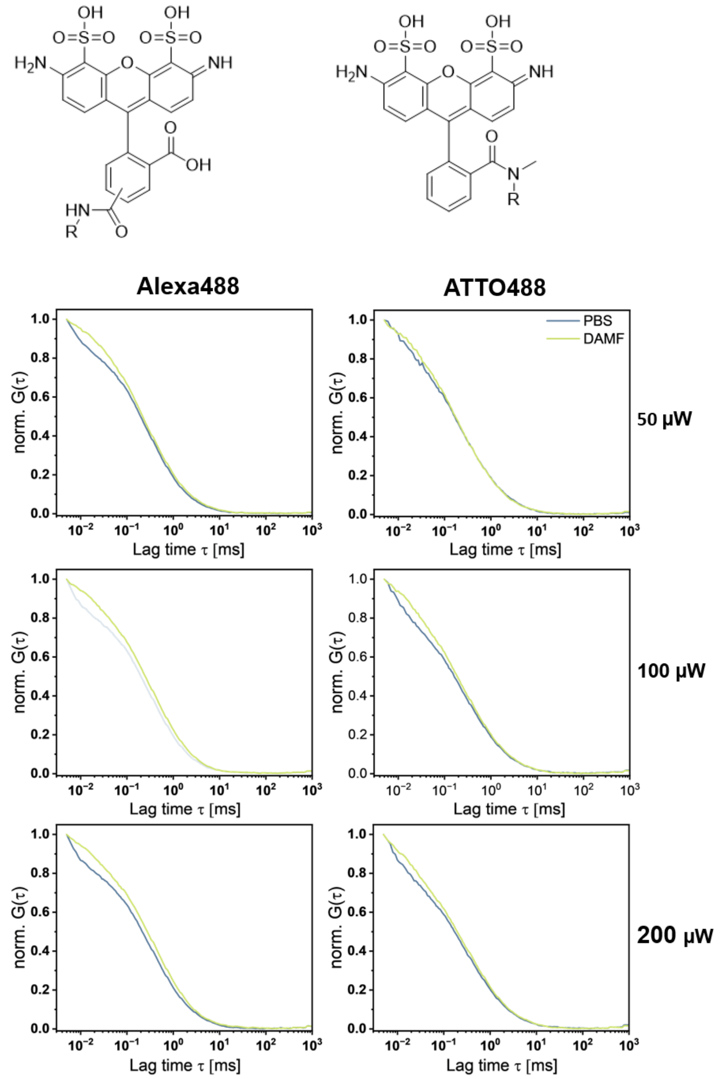

Figure S4: a) Chemical structure of fluorophores Alexa 488 (left) and ATTO488 (right). **b)** FCS power dependence of dsDNA labelled with Alexa488 (left) and ATTO488 (right), excited at 488 nm with 50 µW (top), 100 µW (middle), and 200 µW (bottom). Measurements were performed in buffer only (blue) and in the presence of 100 µM DAMF (yellow) on the FRET-Brick.

Table S1: a) Fit parameters extracted from FCS data recorded on the FRET-Brick shown in Figure S1. t_b = Bunching time; b_a = Bunching amplitude; CPM = Counts per molecule

**Power / µW**	**τ_b [µs]**	**b_a**	**CPM [kHz]**	**τ_b [µs]**	**b_a**	**CPM [kHz]**
**Alexa488**	**PBS**			**DAMF**		
**50**	5.6	0.33	21.6	19.6	0.19	19.3
**100**	5.6	0.37	21.6	19.6	0.18	22.1
**200**	5.6	0.40	18.5	19.6	0.19	22.0
**ATTO488**						
**50**	13.0	0.34	6.9	36.0	0.27	7.0
**100**	13.0	0.36	9.0	36.0	0.28	10.8
**200**	13.0	0.37	9.9	36.0	0.30	12.1

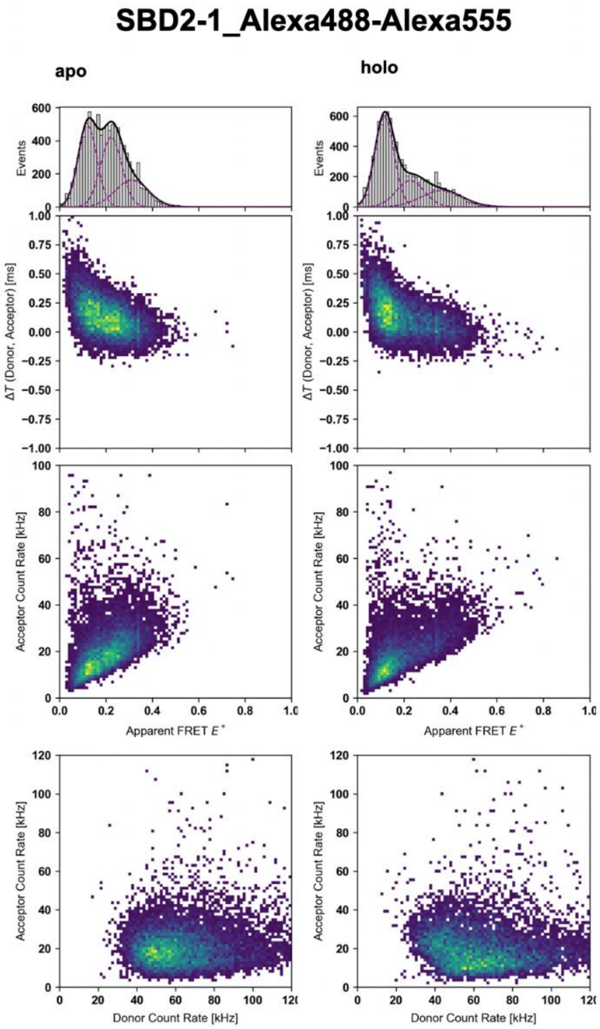

Figure S5: ΔT-E, A-E*, A-D histograms of SBD2 labelled with Alexa 488 (donor) and Alexa555 (acceptor) recorded at 60 kW/cm^2 excitation power with 100 µM DAMF. The left panel shows the apo (open) conformation, the right panel shows the holo (closed) conformation in the presence of 1 mM glutamine.

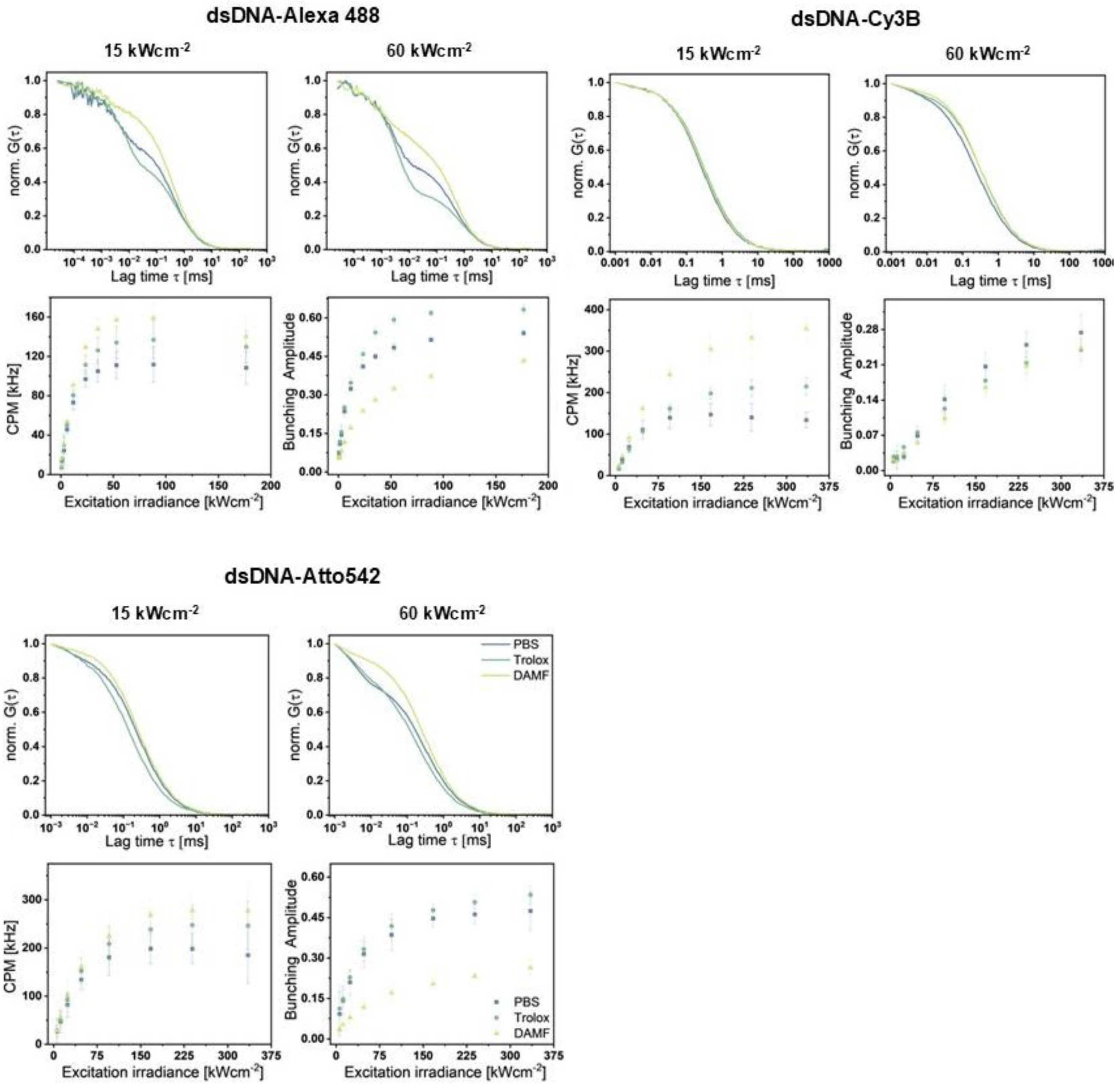

Figure S6: Photostabilizer screening via FCS of Alexa488, Cy3B and Atto542-labeled dsDNA. Upper panels show representative normalized FCS curves at 15 kW/cm^2 (left) and 60 kW/cm^2 (right) excitation power. Buffer conditions are colour-coded: blue, without additives; green, with 1 mM Trolox ((±)-6-hydroxy-2,5,7,8-tetramethylchromane-2-carboxylic acid); and yellow, with 100 µM DAMF ((dimethylaminomethyl)ferrocene). The lower panels summarize the power-dependent screening results for molecular brightness (left) and bunching amplitude (right) to validate the positive photostabilizing effects of Trolox and DAMF.

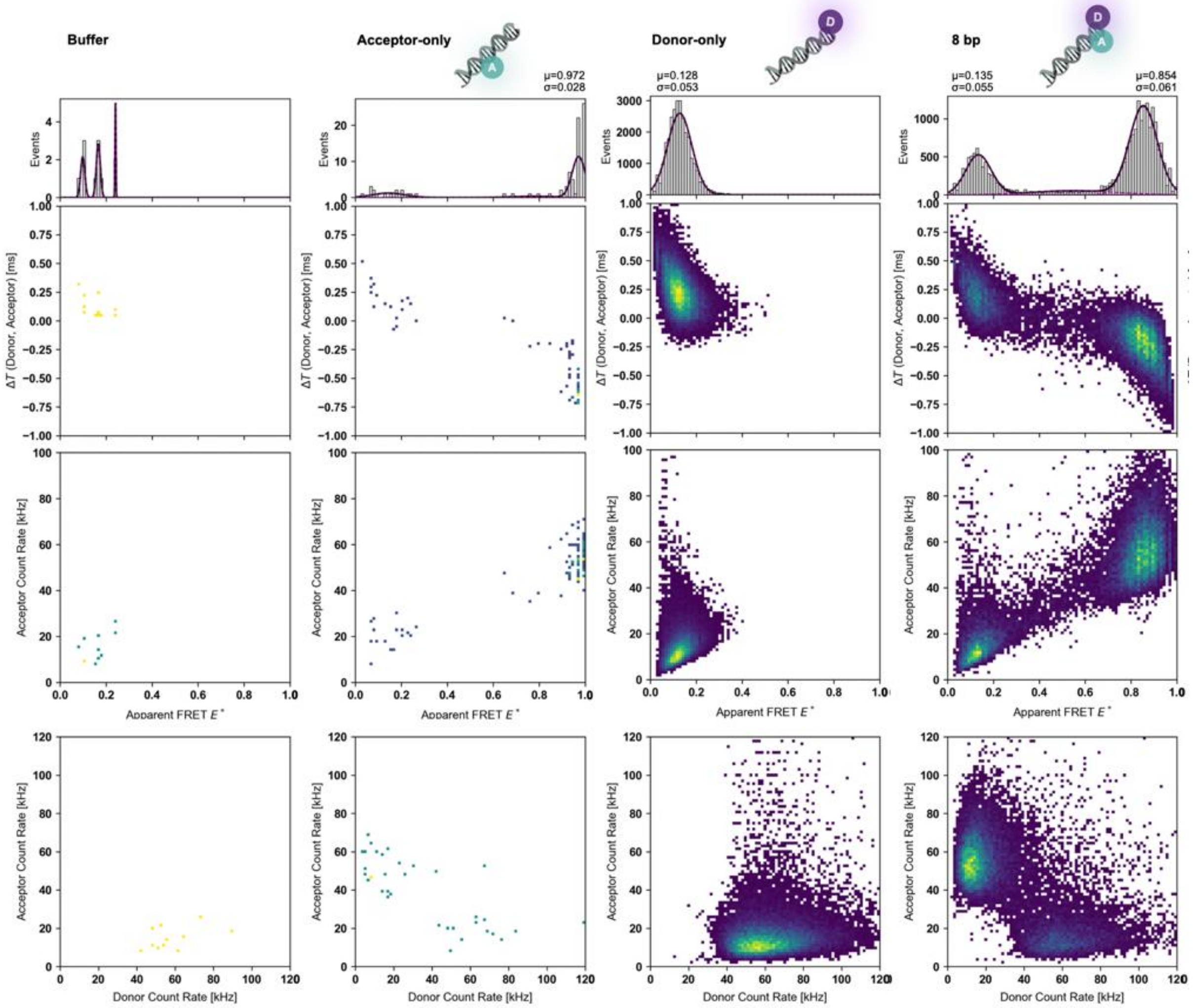

Figure S7: Supporting multiparameter 2D histograms of Alexa 488–Cy3B labeled dsDNA samples. Two-dimensional histograms of a 45-mer dsDNA labeled with Alexa 488 (donor) and Cy3B (acceptor) at 60 kW/cm^2 excitation in the presence of 100 µM DAMF: PBS buffer, acceptor-only, donor-only and donor-acceptor with 8 bp interdye distances. Rows correspond to different parameter spaces: (top) apparent FRET efficiency versus ΔT_{DA}, (middle) apparent FRET efficiency versus acceptor brightness, and (bottom) acceptor versus donor brightness.

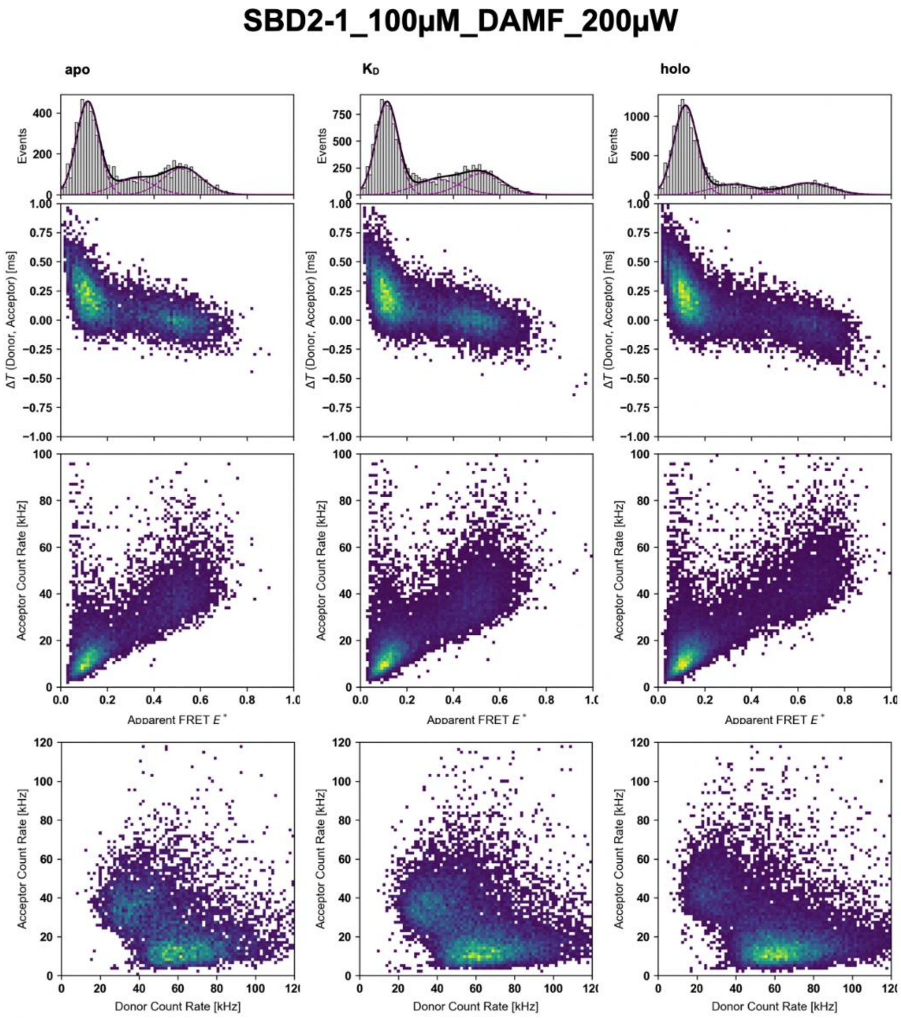

Figure S8: Multiparameter 2D histograms of SBD2 in different ligand-bound states with photostabilizer DAMF. ΔT-E, A-E and A-D histograms SBD2 with Alexa 488 (donor) and Cy3B (acceptor) at 60 kW/cm^2 excitation with 100 μM DAMF. The left panel shows the apo (open), the middle and right panel shows the K_d (2 μM glutamine) conditions and holo state (1 mM glutamine), respectively.

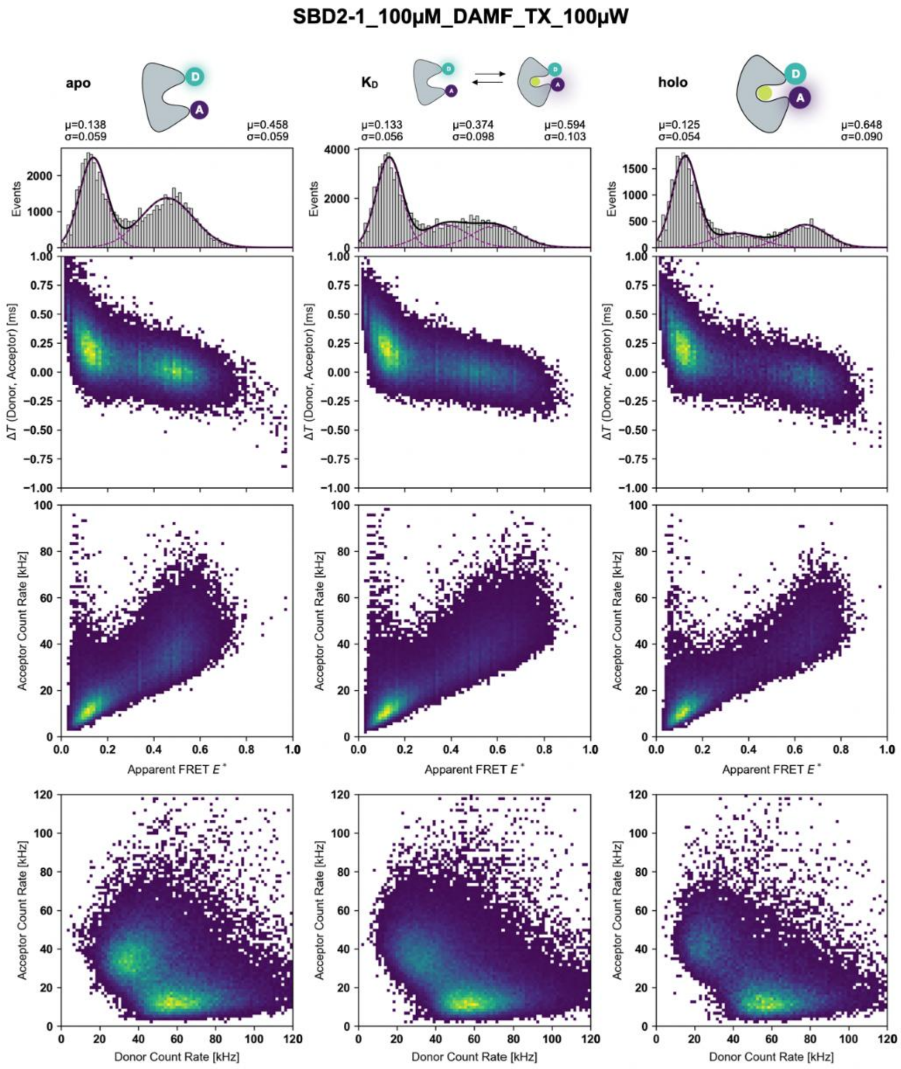

Figure S9: Multiparameter 2D histograms of SBD2 in different ligand-bound states with photostabilizer DAMF and Trolox. ΔT-E, A-E and A-D histograms SBD2 with Alexa 488 (donor) and Cy3B (acceptor) at 60 kW/cm^2 excitation with 100 µM DAMF and 100 µM Trolox. The left panel shows the apo (open), the middle and right panel shows the K_d (2 µM glutamine) conditions and holo state (1 mM glutamine), respectively.

Supplementary Note 1: Material and Methods

General sample preparation

Preparation of labeled DNA: Complementary single-strand fluorophore-labeled oligonucleotides[1] were obtained from Ella Biotech (Fürstenfeldbruck, Germany) with either donor at the 5'-end of the top-strand (Alexa Fluor 488) or acceptor at different positions on the bottom strand (Alexa Fluor 555: 8/18, Cy3b: 8/18 or ATTO 542: 8/18/23 bp; table 1). Double-strand DNAs (dsDNA) were obtained by annealing using the following protocol: A 100-µl solution of two complementary single strand DNA (ssDNA) at a concentration of 1 µM was heated to 95°C for 4 min and then cooled down to 4°C at a rate of 1°C/min in an annealing buffer (20 mM Tris-HCl pH 8.0, 500 mM sodium chloride and 1 mM EDTA). The samples were kept at -20 °C until further usage.

S2 Table: Sequences of DNA strands used in the study.

Strand	Type	Label Role	Sequence
T1	Top strand	Donor	5’-**X**AAATCTAAAGTAACATAAGGTAACATAACGTAAGCTCATTCGCG-3’
B8	Bottom strand	Acceptor	3’-ATTTAGA**X**TTCATTGTATTCCATTGTATTGCATTCGAGTAAGCGC-5’
B18	Bottom strand	Acceptor	3’-ATTTAGATTTCATTGTA**X**TCCATTGTATTGCATTCGAGTAAGCGC-5’
B23	Bottom strand	Acceptor	3’-ATTTAGATTTCATTGTATTCCA**X**TGTATTGCATTCGAGTAAGCGC-5’

***X indicates the position of the fluorophore attached to a T.**

Protein expression and purification: SBD2 double-cysteine variant (T369C-S451C) was expressed and purified as previously described[2]. Briefly, a plasmid containing the coding region, including the appropriate mutations, was transformed into *Escherichia coli* BL21 (DE3) pLysS cells. The transformed cells were grown in 1 liter of LB medium containing 100 µg/mL Kanamycin and 50 µg/mL chloramphenicol at 37 °C under aerobic conditions. Bacterial growth was tracked using absorption measurements at 600 nm wavelength (OD_{600nm}). Overexpression of the protein was induced at an OD_{600nm} of 0.6-0.7 by adding 1 mM IPTG to the culture media, followed by further incubation for 1.5-2.0 hours. Cells were centrifuged at 5000 x g for 30 min at 4 °C (Beckman, JA10). The cell pellet was resuspended in lysis buffer (50 mM Tris-HCl, pH 8.0, 1 M KCl, 10 mM imidazole, 10% glycerol and 1 mM dithiothreitol (DTT)) containing EDTA-free Protease Inhibitor Cocktail (cOmplete™, Roche), and incubated at 4 °C for 1 hour at gentle shaking. Following incubation the cells were disrupted by sonication (Branson tip sonication; amplitude: 25%; 10 min; 0.5 s on-off pulses; tube was kept in ice-water bath to avoid heating) and cell lysate was fractionated by two-step centrifugation (at 4 °C for 30 min at 4,416 g, Eppendorf, Centrifuge 5804 R) and at 4 °C for 1 hour for ultracentrifugation (70,658 g, Beckman, Type 70Ti under vacuum). The protein was purified by affinity chromatography using the Ni^{2+}-Sepharose fast flow resin (GE Healthcare). The supernatant was loaded onto a pre-equilibrated resin and incubated for 1 hour at 4 °C with gentle shaking (resin equilibration included a 10 mL water wash followed by a 40 mL wash with lysis buffer).

The resin-bound protein was washed with 40 ml lysis buffer, followed by 40 ml of wash buffer I (50 mM Tris-HCl, pH 8,0, 50 mM KCl, 20 mM imidazole, 10% glycerol and 1 mM DTT), and finally eluted with 10 ml of elution buffer (50 mM Tris-HCl, pH 8.0, 50 mM KCl, 250 mM imidazole, 10% glycerol and 1 mM DTT). The eluted protein was dialyzed overnight against imidazole-free elution buffer, using Snakeskin™ dialysis membrane, to prevent unfolding/refolding interruptions. The dialyzed sample absorbance was measured at 280 nm, and the sample was aliquoted and stored at -80 °C until further handling.

Unfolding and refolding process of SBD2 (T369C-S451C): The protein was diluted to a concentration of 4 µM in 50 ml of unfolding buffer (10 mM Hepes, pH 7.3 and 6M guanidine hydrochloride) and incubated for 3 hours at 30 °C with gentle shaking. Then, the sample was cooled down and centrifuged (3,046 x g for 30 minutes at 4 ℃) to remove insoluble aggregates prior to the refolding step. The supernatant was transferred to a Snakeskin™ dialysis membrane and dialyzed first against 2 l of L-arginine buffer (200 mM L-arginine, 150 mM NaCl, 10 mM Hepes pH 7.3, 5 mM DTT) for 18 hours, followed with 24 hours dialysis against 5 l dialysis buffer (150 mM NaCl, 10 mM Hepes pH 7.3, 5 mM DTT) with gentle stirring to ensure slow refolding and homogeneity. The refolded protein was then concentrated from 50 mL to a final 500 µL (Vivaspin 10 kDa MWCO; 3,000 g at 4 °C) and further purified by size-exclusion chromatography (ÄKTA pure system, Superdex-75 Increase 10/300 GL, GE Healthcare) and stored at -80 °C with 5% glycerol until further handling.

Protein labeling: The refolded SBD2 (T369C-S451C) was labelled as previously published[2,3] using Alexa Fluor 488, Alexa Fluor 555 and Cy3B. Briefly, 0.6 mg of SBD2 (T369C-S451C) were incubated with 10 mM DTT ion labelling buffer (50 mM Tris-HCl pH 7.6, 150 mM NaCl) for 1 hour at 4 ℃. After incubation, the DTT concentration was reduced to 5 mM, and the protein sample was immobilized on 200 µL of Ni^{2+}-Sepharose resin that had been washed and pre-equilibrated with labeling buffer. The resin was washed with 36 ml of labelling buffer followed by an overnight incubation with the fluorophores solution at 4 ℃ (50nmol of each fluorophore, dissolved in 0.7 ml of labelling buffer). Following incubation, excess unreacted fluorophores were removed by washing with 12 ml of labeling buffer, and the protein was eluted with 0.5 ml of labeling buffer containing 500 mM imidazole. The labelled protein was then purified by anion-exchange chromatography (AIEX) on an ÄKTA pure chromatography system (Cytiva; MonoQ 5/50 GL column, Cytiva) as previously described[4].

FCS-Guided Photostabilizer Screening Photostabilizers

Sample handling: DNA duplexes containing a single fluorophore were diluted to 0.5–1 nM in 100 µL PBS and measured on coverslips passivated with 1 mg/mL BSA in a series of 5–10 measurements each lasting 1 min. Photostabilizers were added to final concentrations of 1 mM Trolox ((±)-6-Hydroxy-2,5,7,8-tetramethylchromane-2-carboxylic acid, Sigma-Aldrich) or 100 µM DAMF ((Dimethylaminomethyl)ferrocen, Sigma-Aldrich).

485-nm excitation (FCS of Alexa Fluor 488): FCS experiments of Alexa Fluor 488 labeled dsDNA were conducted on an inverted microscope (MicroTime 200, PicoQuant) equipped with single photon counting electronics and picosecond time resolution (Hydra Harp 400, PicoQuant) or the FRET-Brick (see below). The sample was excited either through a 60x water immersion objective (Nikon M Plan Apo NA 1.20) or a 60x oil

immersion objective (Olympus UPlanSApo NA 1.2) to in a diffraction-limited spo. The light emitted by the sample was collected through the same objective and split into perpendicular/parallel (polarizing beam splitter, PBS) components and detected by single-photon avalanche detectors (SPCM-AQR-14, Perkin Elmer) through green (BrightLine HC 520/35) band-pass filters. A linearly polarized laser diode (LDH-D-C-485 with 485 nm, PicoQuant) operated in continuous-wave mode excited the sample.

532-nm excitation (FCS of Atto 542 and Cy3B): For FCS experiments in the green spectral range, we used a custom-made ALEX microscope[3]. An OBIS 532-100-LS laser (Coherent, USA) provided continuous-wave excitation. The laser beam is coupled into a polarization-maintaining single-mode fiber (P3-57-488PM-FC-2, Thorlabs) via an aspheric fiber port (PAF2S-11A, Thorlabs), collimated (RC12APC-P01, Thorlabs) and guided into the epi-illuminated confocal microscope (Olympus IX71, Hamburg, Germany) by dual-edge beamsplitter ZT532/640rpc (Chroma/AHF) focused by a water immersion objective (UPlanSApo 60x/1.2w, Olympus Hamburg, Germany). The emitted fluorescence is collected through the objective and spatially filtered using a pinhole with 50 µm diameter and spectrally split into donor and acceptor channel by a single-edge dichroic mirror H643 LPXR (AHF). Fluorescence emission was filtered (donor: BrightLine HC 582/75 (Semrock/AHF), acceptor: Longpass 647 LP Edge Basic (Semrock/AHF), focused on avalanche photodiodes (SPCMAQRH-64, Excelitas). The detector outputs were recorded by a counter/timer device module (USB-CTR04, Measurement Computing, USA) using custom-made acquisition software written in Python, available as a compiled executable or editable code at https://github.com/harripd/mcc-daq-acquisition.

The FRET-Brick

Optical configuration for the FRET-Brick (see also Figure 1a, main text): For the excitation pathway, continuous-wave (CW) excitation was supplied by a USB-powered blue laser diode (488-30-1235-BL, Q-LINE). The laser power was adjusted through a continuous neutral density filter wheel (NDC-50C-2M, Thorlabs) and guided into an inversely mounted reflective collimator (RC08FC-P01, Thorlabs, USA), which coupled the beam into a polarization-maintaining single-mode fiber P3-57 488PM-FC-2 (Thorlabs, USA). The fiber guided the beam into the excitation layer, where it was collimated (RC12APC-P01, Thorlabs, USA). A dichroic beamsplitter with high reflectivity at 488 nm (ZT491rdc, Chroma/AHF, Germany) separated the excitation and emission beams to and from a high-NA apochromatic objective (60×, NA 1.2, UPlanSAPO 60XW, Olympus, Japan). For the emission and detection pathway, the emitted fluorescence was collected by the same objective and directed via a mirror into a piezo-directed optical mount (AG-M100N, Newport). The beam passed through an inversely mounted 12-mm reflective collimator (RC12FC-P01, Thorlabs), which focused and coupled the emission into a multimode optical fiber (10-µm core diameter, M64L01, Thorlabs). The fiber delivered the emission to a detection box, where it was collimated by a fixed-focus collimator (F220FC-532, Thorlabs) and spectrally split into two photon streams by a dichroic mirror (ZT543rdc longpass, Chroma/AHF, Germany). Individual photon streams were filtered with a band-pass filter for the donor channel (FF03-525/50, Semrock, Rochester NY, USA) and, for the acceptor channel, a 488-nm notch filter (NF488-15, Thorlabs, USA), and detected by PMTs (H10682-210, Hamamatsu, Japan). The detector outputs for FCS analysis were recorded via a counter/timer device module (USB-CTR04, Measurement Computing, USA) using custom-made acquisition software written in Python, available as a compiled executable or editable code at https://github.com/harripd/mcc-daq-acquisition.

3D printing and assembly of the FRET-Brick: The FRET-Brick was assembled using components from previously printed parts for the μFCS and μALEX modality of the Brick-MIC[4], which required no new parts to be printed. Specifically, the excitation layer of the μALEX modality was combined with the emission layer and detection box of the μFCS modality. All models were described in detail in ref. [4], which was designed and conceived using Onshape versions 1.114 to 1.172. 3D printing was carried out using PLATech filament (OLYMPfila) on an Ultimaker +2 Extended fitted with a 0.4-mm nozzle. All models were printed with an infill density of 17%, three layers for outer walls and with a layer height of 0.1 mm. The printing speed was set to 50 mm/s, and the nozzle temperature was maintained at 210°C. To prevent warping, all parts were printed with a brim and without any supports.

Sample handling: FRET-labeled 45-mer dsDNA samples were measured at a concentration of 100 pM in 100 µl droplets placed on pre-passivated coverslips (1 mg/ml BSA in PBS buffer), with or without the addition of the respective photostabilizers as detailed in the text and figure captions. The sample was excited at 488 nm with an excitation power of 200 µW (measured before entering the objective). Each measurement lasted on average 30 minutes.

Protein samples were measured at 100 pM in 100 µl droplets of 50 mM Tris-HCl pH 7.6, 150 mM NaCl buffer on pre-passivated coverslips (1 mg/ml BSA in PBS buffer). The sample was excited at 488 nm with an excitation power of 100 µW (measured before entering the objective). Proteins were recorded under three conditions: without ligand (apo), saturated with 1 mM glutamine (holo), and at an intermediate ligand concentration of 2 µM glutamine (approximately K_d). Each condition was measured with 100 µM DAMF and 100 µM Trolox. Measurements lasted on average 45 minutes.

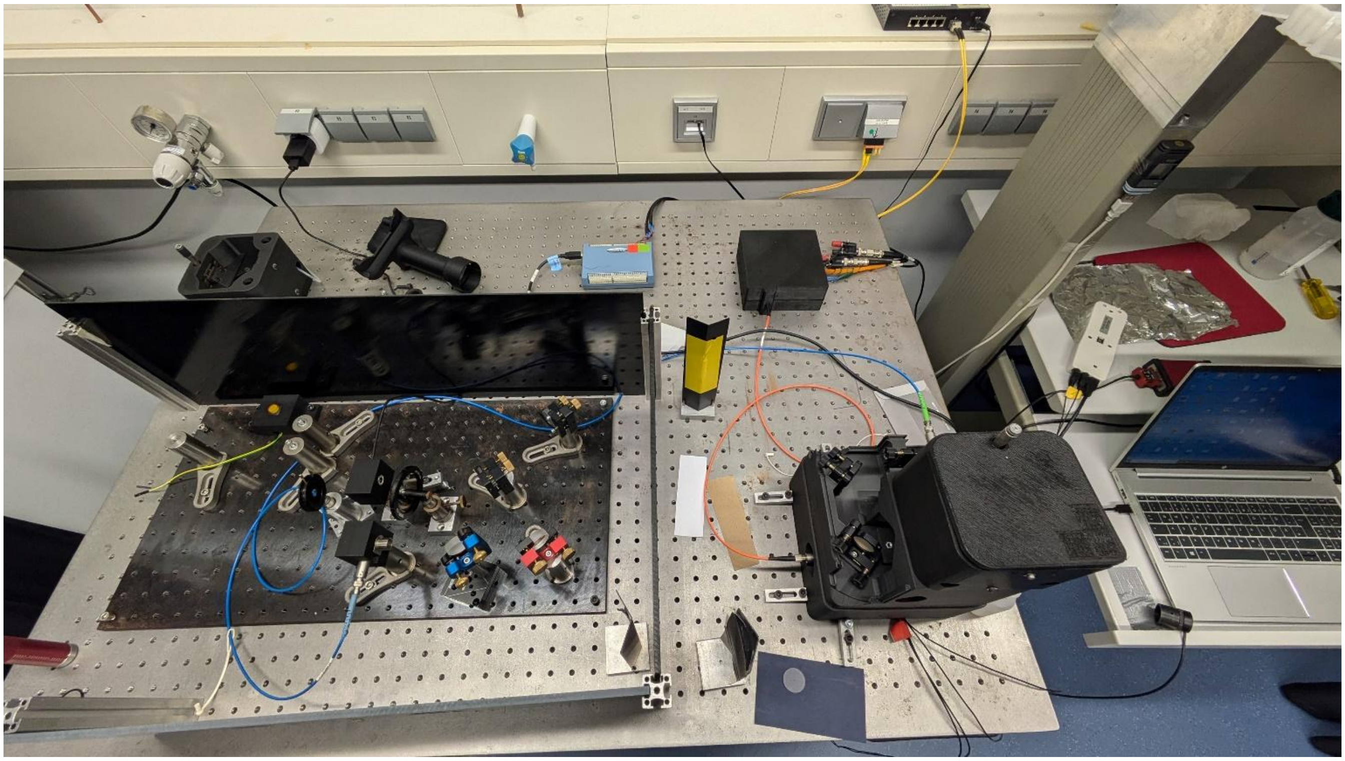

Figure S10: Photograph of the FRET-Brick.

Supplementary Note 2: Single-molecule data analysis

Burst analysis

The used approaches to analyze photons within a burst (burst-wise analysis) groups are implemented in freely available open-source software[5,6]. Briefly, for every burst we determine spectroscopic parameters, such as channel, X, dependent integrated photon counts, n_X, corresponding signal intensities, S_X, apparent FRET efficiency E* and background, cross-talk and sensitivity-corrected FRET efficiencies, E,[7] and the average burst pixel arrival times, T_X. We determine these parameters for different emission channels: referred to as G refers to the "blue" and R refers to "green". The subscript "…|G" indicates excitation of the sample with a blue light source. The blue and green detection channels are denoted by the subscripts G|…, and R|…. The subscript "R|G" denotes green detection following blue excitation. The molecular species are indicated by superscripts; for instance, (DA) refers to a species. In the burst-wise single-molecule analysis, we group the photon stream into individual bursts by discriminating the background (approximately 1-2 kHz) from the fluorescence signal by applying an intensity threshold criterion[8]. Here, we use the total signal recorded for G|G and R|G to discriminate photons from the background. The background, the detection efficiency-ratio of the "blue" and "green" detectors, and the spectra were considered to determine the FRET efficiency, E, of every burst[7]. Following the grouping of photons, we determine for every photon group/pixel the signal intensities $S_{B|B}$ and $S_{G|B}$ to compute an apparent FRET efficiency E*:

$$E^* = \frac{S_{R|G}}{S_{R|G} + S_{G|G}} \qquad \textbf{(1)}$$

In addition to E* we compute $\Delta T(G,R)$, the difference between the duration of a burst when only inspecting $G|G$ or $R|G$ photons:

$$\Delta T(G,R) = dur(G|G) - dur(R|G) \qquad \textbf{(2)}$$

This additional burst metric makes use of time and channel information and identifies bursts, where the acceptor bleached within the burst duration.

Photon distribution analysis

To maximize the analysis precision of our single molecule experiments by explicitly considering the photon shot-noise of the data we used a Photon Distribution Analysis[9] model implemented in the software ChiSurf[10]. The used model explicitly accounts for FRET, excitation cross talks (direct acceptor excitation), detector efficiencies, spectral crosstalks, and background contributions. Heterogeneities that are likely associated acceptor-induced histogram broadening beyond the shot-noise[11] was modelled by a mixture of Gaussian distance distributions, which mapped to a distribution of FRET efficiencies. The parameters describing the fluorescence quantum yield and spectra were determined separately.

Briefly, the donor-acceptor distance distribution was modeled as normalized mixture of K Gaussians.

$$p(R_E) = \sum_{k=1}^{K} a_k N(r|\mu_k, \sigma_k), \quad \sum_{k=1}^{K} a_k = 1 \qquad \textbf{(3)}$$

Where R_E is a distance, associated to the FRET efficiency, μ_k, σ_k, and a_k denote mean distance, width and amplitude of the k-th component, respectively. The corresponding FRE efficiency was obtained via:

$$E(R_{DA}) = \frac{1}{1+\left(\frac{R_E}{R_0}\right)^6}. \qquad \textbf{(4)}$$

The distance distribution was discretized on a linear distance scale from 0.5 to 15.0 nm, resulting in a discretized $p(E_i)$ that is used in later steps to compute model FRET histograms. To account for excitation and emission crosstalks we first compute the fluorescence, intensity matrix, $\mathbf{I}$

$$\mathbf{I} = \begin{bmatrix} I_{G|G} & I_{G|R} \\ I_{R|G} & I_{R|R} \end{bmatrix} = \mathbf{DFX} \qquad \textbf{(5)}$$

Where $\mathbf{X}$ is the excitation matrix dependent onf the light source, L

$$\mathbf{X} = \begin{bmatrix} \sigma_{D|G} L_G & \sigma_{D|R} L_R \\ \sigma_{A|G} L_G & \sigma_{A|R} L_R \end{bmatrix} \qquad \textbf{(6)}$$

and $\sigma_{d|l} L_s$, the absorption of the fluorophore $d \in \{D, A\}$ excited by the light source $s \in \{R, G\}$. Here, we performed OCE. Hence, L_R=0. In $\mathbf{X}$, off-diagonal elements are excitation cross-talks. Depending on the FRET efficiency, E, the intensities are redistributed across the emission channels.

$$\mathbf{F} = \begin{bmatrix} (1-E) & 0 \\ E & 1 \end{bmatrix} \qquad \textbf{(7)}$$

before being detected.

The detection is described by, $\mathbf{D}$**,** the detection matrix that accounts for fluorescence quantum yields of the donor $\Phi_{F,D}$ and acceptor, $\Phi_{F,D}$, respectively,

$$\mathbf{D} = \begin{bmatrix} g_{G|D} \Phi_{F,D} & g_{G|A} \Phi_{F,A} \\ g_{R|D} \Phi_{F,D} & g_{R|A} \Phi_{F,A} \end{bmatrix}, \qquad \textbf{(8)}$$

the spectral sensitivities $g_{c|s}$ for the spectral channel, $c \in \{G, R\}$, and the species, $s \in \{A, D\}$. Off-diagonal elements are emission cross-talks.

Using the elements of the fluorescence intensity matrix, $\mathbf{I}$, p_G, the probability of the model for detecting a photon in the "Green" detection channel is:

$$p_G = \frac{I_{G|G}(E)}{I_{G|G}(E)+I_{R|G}(E)}. \qquad \textbf{(9)}$$

Given, p_G, the conditional probability of observing a particular combination of green and red fluorescence photons, F_G and F_R probability, $P(F_G, F_R\,|F)$, for F registered fluorescence photons follows a binomial distribution:

$$P_M(F_G, F_R|F) = \frac{F!}{F_G!(F-F_G)!} p_G^{F_G} (1-p_G)^{F-F_G}. \quad \textbf{(10)}$$

Here, we accounted for the background by modelling the background signals B_G and B_R by Poisson distributions, $P(B_G)$ and $P(B_R)$ to obtain the probability for a particular combination of fluorescent photons, $P_M(S_G, S_R)$:

$$P_M(S_G, S_R) = \sum_{F_G+B_G=S_G; F_R+B_R=S_R} P(F) P_M(F_G, F_R|F) P(B_G) P(B_R). \quad \textbf{(11)}$$

$P(F)$ is the fluorescence intensity distribution, that is obtained from the total signal intensity distribution $P(S)$. To optimize model parameters such as a_k, μ_k or σ_k we either score the modelled $P_M(S_G, S_R)$ or $P_M(S_G, S_R)$ marginalized to E_{app} against experimental histograms. The analysis procedure was implemented in the open-source software ChiSurf (https://github.com/fluorescence-tools/chisurf).

<u>Fluorescence correlation spectroscopy (FCS)</u>

Data analysis: Photon streams were binned to generate intensity traces and autocorrelated with a multi-tau algorithm[12] implemented in the software Chisurf[10] (https://github.com/fluorescence-tools/chisurf). The obtained autocorrelation functions, $G(\tau)$, were fitted to a 3D Brownian diffusion model:

$$G_d(\tau) = \frac{1}{N} \cdot \left(1 + \frac{\tau}{t_d}\right)^{-1} \cdot \left(1 + \frac{1}{s^2} \cdot \frac{\tau}{t_d}\right)^{-0.5}. \quad \textbf{(12)}$$

Here, N, τ, t_d, and s are the effective number of molecules, the correlation time, the diffusion time, and a structure factor of the confocal volume, respectively.

Alexa Fluor 488 was described by a single dark-state relaxation term:

$$G(\tau) = G_d(\tau) \cdot \left(1 - b_a + b_a \cdot \exp\left(-\frac{\tau}{b_t}\right)\right) + b. \quad \textbf{(13)}$$

Experiments with Atto 542 and Cy3B were described by two dark-state relaxation terms:

$$G(\tau) = G_d(\tau) \cdot \left(1 - b_{a,1} + b_{a,1} \cdot \exp\left(-\frac{\tau}{b_{t,1}}\right) - b_{a,2} + b_{a,2} \cdot \exp\left(-\frac{\tau}{b_{t,2}}\right)\right) + b \quad \textbf{(14)}$$

Above, b, b_a, b_t, are baseline offset, triplet amplitude, triplet lifetime, diffusion time, structure parameter, number of molecules and triplet relaxation term respectively.

SI References

[1] E. Ploetz, E. Lerner, F. Husada, M. Roelfs, S. Chung, J. Hohlbein, S. Weiss, T. Cordes, “Förster resonance energy transfer and protein-induced fluorescence enhancement as synergetic multi-scale molecular rulers” *Sci Rep* **2016**, *6*, 33257.

[2] G. Gouridis, G. K. Schuurman-Wolters, E. Ploetz, F. Husada, R. Vietrov, M. de Boer, T. Cordes, B. Poolman, “Conformational dynamics in substrate-binding domains influences transport in the ABC importer GlnPQ” *Nat Struct Mol Biol* **2015**, *22*, 57–64.

[3] M. de Boer, G. Gouridis, R. Vietrov, S. L. Begg, G. K. Schuurman-Wolters, F. Husada, N. Eleftheriadis, B. Poolman, C. A. McDevitt, T. Cordes, “Conformational and dynamic plasticity in substrate-binding proteins underlies selective transport in ABC importers” *eLife* **2019**, *8*, e44652.

[4] G. G. Moya Muñoz, O. Brix, P. Klocke, P. D. Harris, J. R. Luna Piedra, N. D. Wendler, E. Lerner, N. Zijlstra, T. Cordes, “Single-molecule detection and super-resolution imaging with a portable and adaptable 3D-printed microscopy platform (Brick-MIC)” *Sci. Adv.* **2024**, *10*, eado3427.

[5] T.-O. Peulen, K. Hemmen, A. Greife, B. M. Webb, S. Felekyan, A. Sali, C. A. M. Seidel, H. Sanabria, K. G. Heinze, “tttrlib: modular software for integrating fluorescence spectroscopy, imaging, and molecular modeling” *Bioinformatics* **2025**, *41*, btaf025.

[6] T.-O. Peulen, O. Opanasyuk, C. A. M. Seidel, “Combining Graphical and Analytical Methods with Molecular Simulations To Analyze Time-Resolved FRET Measurements of Labeled Macromolecules Accurately” *J. Phys. Chem. B* **2017**, *121*, 8211–8241.

[7] E. Sisamakis, A. Valeri, S. Kalinin, P. J. Rothwell, C. A. M. Seidel, “Accurate single-molecule FRET studies using multiparameter fluorescence detection” *Methods Enzymol* **2010**, *475*, 455–514.

[8] C. Eggeling, S. Berger, L. Brand, J. R. Fries, J. Schaffer, A. Volkmer, C. A. M. Seidel, “Data registration and selective single-molecule analysis using multi-parameter fluorescence detection” *Journal of Biotechnology* **2001**, *86*, 163–180.

[9] S. Kalinin, S. Felekyan, M. Antonik, C. A. M. Seidel, “Probability Distribution Analysis of Single-Molecule Fluorescence Anisotropy and Resonance Energy Transfer” *J. Phys. Chem. B* **2007**, *111*, 10253–10262.

[10] T.-O. Peulen, “Exploring Time-Resolved Fluorescence Data: A Software Solution for Model Generation and Analysis” *Spectroscopy Journal* **2025**, *3*, 16.

[11] S. Kalinin, E. Sisamakis, S. W. Magennis, S. Felekyan, C. A. M. Seidel, “On the Origin of Broadening of Single-Molecule FRET Efficiency Distributions beyond Shot Noise Limits” *J. Phys. Chem. B* **2010**, *114*, 6197–6206.

[12] S. Felekyan, R. Kühnemuth, V. Kudryavtsev, C. Sandhagen, W. Becker, C. A. M. Seidel, “Full correlation from picoseconds to seconds by time-resolved and time-correlated single photon detection” *Review of Scientific Instruments* **2005**, *76*, 083104.
